# Computer Simulation of the Mechanical Behavior of the ‘Zygomatic Bones–Implants–Splinting Bar–Removable Overdenture’ Dental Structure Under Operational Loads

**DOI:** 10.3390/dj13090393

**Published:** 2025-08-28

**Authors:** Magomed Magomedov, Alexander Kozulin, Sergey Arutyunov, Alexey Drobyshev, Timur Dibirov, Eduard Kharazyan, Magomet Mustafaev, Artem Drobyshev, Sergey Panin

**Affiliations:** 1 Maxillofacial Surgery Department, Russian University of Medicine, 127006 Moscow, Russia; magomedovich001@yandex.ru (M.M.); dr.drobyshev@gmail.com (A.D.); rumit.05@mail.ru (T.D.); edwardkharazian@hotmail.com (E.K.); drobartem459@gmail.com (A.D.); 2 Department of Mechanics of Solids, National Research Tomsk State University, 634050 Tomsk, Russia; kozulyn@ftf.tsu.ru; 3 Digital Dentistry Department, Russian University of Medicine, 127006 Moscow, Russia; sd.arutyunov@mail.ru; 4 Institute of Dentists and Maxillofacial Surgery, Kabardino-Balkarian State University, 360004 Nalchik, Russia; musmag@mail.ru; 5 Laboratory of Mechanics of Polymer Composite Materials, Institute of Strength Physics and Materials Science of Siberian Branch of Russian Academy of Sciences, 634055 Tomsk, Russia

**Keywords:** dental structure, failure, finite element method (FEM), removable overdenture, splinting bar, stress-strain state (SSS), zygomatic implant

## Abstract

**Background/Objectives:** When solving the problems of installing zygomatic implants after partial or full maxillectomy with subsequent attachment of a removable overdenture (ROD), computer simulation based on the finite element method (FEM) is an effective tool for treatment planning. In this study, stress-strain states of the ‘zygomatic bones–implants–splinting bar–ROD’ dental structure were evaluated under various loading conditions. **Methods:** A 3D FEM computer simulation was carried out to estimate stress-strain states of the elements of the dental structure and to study the effect of redistribution of the loads transferred from the ROD to the zygomatic bones through four implants. **Results:** That successive insertion and removal of the ROD caused identical stresses in the elements of the dental structure. Given the accepted level of critical stress of about 13 MPa, their values may be exceeded in the zygomatic bones during both processes. In the ROD, the equivalent stresses did not exceed the critical levels upon alternate loading of 50 N on the posterior teeth (both molars and premolars) under all biting and mastication. Taking into account the linear dependence of the applied load and the stresses in the ROD, it can be stated that its integrity is maintained until 118 N (or the generally accepted typical value of 100 N). Under the 90° biting angle, the equivalent stresses are below the critical level in all the studied cases; thus, the acceptable value increases to 213 N, but it is only 63 N at a biting angle of 45°. **Conclusions:** It has been established that the equivalent stresses in the zygomatic bones can exceed the critical stress level of 13 MPa. In addition, some practical recommendations and prospects of the study have been formulated.

## 1. Introduction

Currently, the design and implementation of new diagnostic processes, pathogenetic treatment, and restoration of the function and aesthetics of the oral cavities of patients with personalized consideration of the rehabilitation processes are of particular importance. Zygomatic implants are a viable solution for the rehabilitation of patients with cancer, especially when bone grafting is contraindicated or inappropriate. In particular, combined metal-polymer dental structures are widely used [1].

However, failures in rehabilitation are inevitable and are most often associated with loosening (disintegration) of implants [2,3]. In such cases, the main reason is the occlusal overload of both teeth and dental arches, which can lead to microfractures of bones at the interfaces with the implants. In addition, stresses may be insufficient for stable retention of high-stiffness implants that absorb loads but do not stimulate the bone growth around them. This is because of their incompatibility with bone tissue, both mechanically and biologically.

After installation, zygomatic implants are in tight contact with the bone tissue without any compensations or redistributions of loads on the periodontal ligament, which could partly absorb the loads [4]. General somatic conditions of the bone tissue of patients requiring implant placement are an important factor, since osteoporosis often occurs in such cases. As a result, they are poorly osseointegrated, delaying prosthetics. Therefore, the development of approaches to peri-implant biomechanics with detailed patient characteristics at the operation planning stage will promote adequate osteoimmunomodulatory reactions. In addition, a balance between osteoblastic and osteoclastic activity must be achieved to ensure bone homeostasis and long-term implant stability [5,6,7,8].

When solving practical problems related to the installation of four zygomatic implants after partial or full maxillectomy and subsequent attachment of a removable overdenture (ROD), computer simulation based on the finite element method (FEM) is an effective tool for treatment planning [9,10]. In particular, it is used to assess the stress-strain states (SSSs) of both engineering and biomechanical structures [11,12]. Some advantages lie in the non-invasive nature of the process and the possibility of theoretically estimating the stresses developing in all regions.

Such computer simulations are motivated by the necessity to assess the maximum stresses and strains in the regions of attachment of implants to the zygomatic bones, with the possibility of adjusting the number, size, and locations of implants. One of the reasons for this ambiguity is the different conditions (density) of the bone tissue in the potential installation region of each implant chosen by a surgeon [13]. In addition, the zygomatic bones may perceive loads from the ROD through high-strength titanium implants differently due to their possible graded structures (for example, cortical and spongy tissues) and varied connections with other skull bones. Finally, the ROD design may assume the possibility of their independent insertion and detachment by a patient (in contrast to non-removable structures rigidly fixed to abutments). In this case, a splinting bar with metal or polymer clamps is integrated into the dental structure [14]. Upon its removal, loads can be transmitted to the zygomatic bones at levels exceeding the tissue strength, causing failure or disintegration.

In the context of planning prosthetic treatment with the installation of implants, the SSSs of bone tissue should be analyzed, primarily in the regions of their attachment. The authors have previously conducted similar studies [15], which demonstrated the potential of computer simulations. Its key advantage is not the exact reproduction of all anatomical features of the zygomatic bones and skull as a whole, based on the computed tomography (CT) data. However, the reliability of the predicted maximum stresses and strains in the components of a dental structure is questionable.

As shown in [16,17], stabilization of the zygomatic bones with zygomas in two areas and the alveolar ridge with transverse splinting is important for ensuring stiffness of dental structures, while resorting to other fixation options is not recommended at all [18]. Nevertheless, severe options should be carefully considered when the alveolar ridge is absent. In doing so, fixation with the zygomas is possible only in the zygomatic bones at the top end of the implants.

In this study, the SSSs of the ’zygomatic bone–implant–splinting bar–ROD’ dental structure were evaluated under various loading conditions. The splinting bar serves two functional purposes: it splints all four zygomas and serves as a basis for fixing the ROD with clamps. Its application allows for the redistribution of stresses transmitted from the ROD on the one hand. On the other hand, it can cause excessive concentration of stress upon non-coaxial loading, which can result in fracture of the zygomatic bones in the implant attachment regions.

The remainder of this paper is organized as follows. Section 2 describes the problem statement, computer simulation methodology, and model applied. Section 3 is devoted to the characterization of the obtained results. Conclusions are preceded by a discussion, generalizing the obtained results and outlining prospects for the development of this scientific direction. Supplementary Files S1 and S2 contain tables of the maximum equivalent stresses in the elements of the dental structure and illustrations of their distributions corresponding to each loading case.

## 2. Research and Computer Simulation Methods

**Figure 1:**
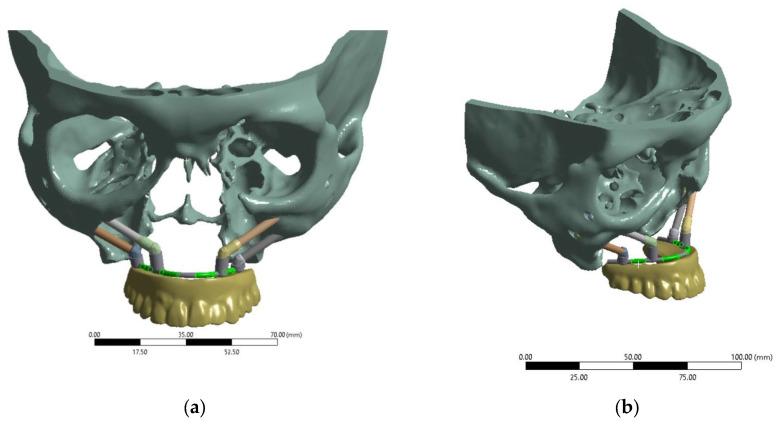
3D model of the ‘zygomatic bones–implants–splinting bar–ROD’ dental structure: (**a**) front (face) view; (**b**) isometric view.

For computer simulation, a 3D model of the ‘zygomatic bones–implants–splinting bar–ROD’ dental structure was developed (Figure 1, hereinafter referred to as the ‘dental structure’). In this way, a skull was reconstructed from the CT data of a real patient using the ‘InVesalius 3.1’ software package [19,20]. The CT data were converted from the ‘DICOM’ format into the ‘STL’ format, which is supported by many CAD applications. Regardless, the ethical standards are not affected (since personal information, photographs of the patient, or any indirect data that would indicate belonging to a specific person are not provided), and the corresponding approval is given at the end of the paper.

**Figure 2:**
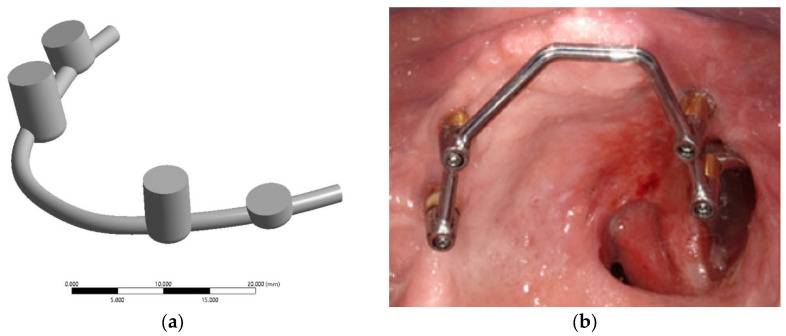
3D model of the splinting bar (**a**) and an example of its real general view (**b**) [21].

Figure 2a presents a model of the splinting bar developed by analogy with the clinical case described in [21], Figure 2b. Its shape was formed for attachment to the installed implants in accordance with the digital model of the skull, with four implants used in a previous paper by the authors [15]. In this study, a virtual ROD model was used. The splinting bar is rigidly attached to the abutments of four implants on one side, but to the ROD with clamps on the other side [22]. Figure 2b presents an example of such a splinting bar that is rigidly attached to the implants with screws. This does not correspond to the maxillectomy case shown in Figure 1.

**Figure 3:**
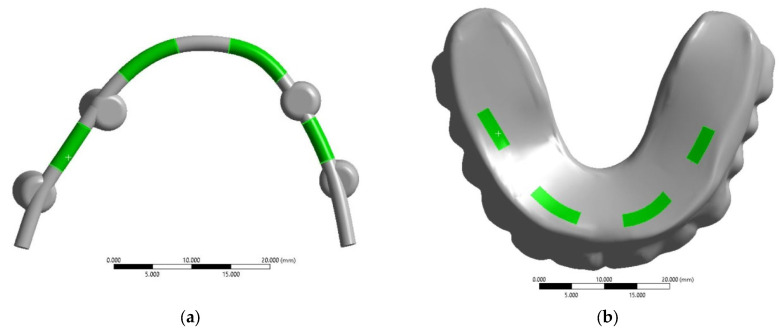

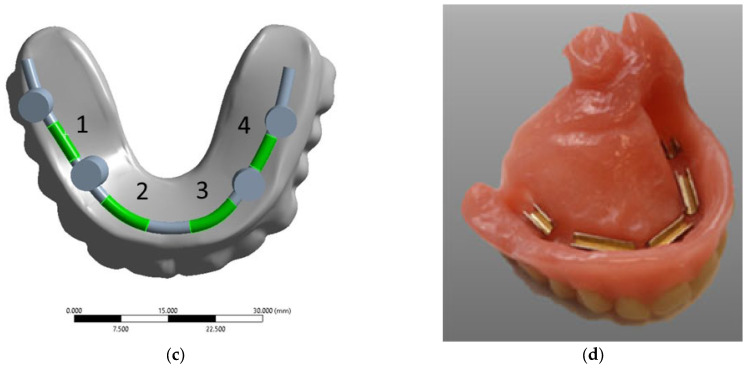
Contact surfaces for the kinematic connection of the splinting bar and the ROD (**a**,**b**); the regions of fixation of the ROD base to the splinting bar by serial snapping along retention zones No. 1–4 (**c**); an example of the placement of clamps in the ROD base (**d**) [21].

In this study, the splinting bar was simulated as a circular-section arch (2 mm in diameter) with four cylindrical sockets, which are rigidly fastened together at the manufacturing stage. The polymer ROD is assumed to be attached to the splinting bar using special clamps (Figure 3d).

The developed model imitates bar (rod) structures used in dental treatment. The male part of the lock is a bar connecting the abutments of osseointegrated zygomatic implants. The female part is inserted and fixed into the ROD, corresponding to the shape of the splinting bar (four clamps in the studied case). The splinting bar includes a matrix (thickening) that is installed on the male (abutment) of the implants, symmetrically positioning them on both sides of the jaw. The splinting bar is attached to the implants’ abutments using titanium pins.

According to specialized medical literature and manufacturers’ information, clamps for fastening a ROD should provide a given level of retention (estimated by the load level required for its removal) and stabilization. In this study, the maximum retention level of 1.8 kg (Medium Retention–Retentive yellow clip) [23] was used as a boundary condition in computer simulation of insertion and removal of the ROD.

The main purpose of computer simulation was to estimate SSSs of the elements of the dental structure and to study the effect of redistribution of the loads transferred from the ROD to the zygomatic bones through four implants. For this reason, the clamp design was idealized in the 3D model (Figure 3). Thus, it is assumed that sockets (fastening holes in the splinting bar) are ‘filled’ with screws. The potential presence of threads was also not taken into account since they do not contribute to SSSs of the elements of the dental structure, but can significantly complicate computer simulation due to local refinement of the FEM mesh. The implant surface was assumed to be fully conjugated over the entire interface with the bone tissue.

The connection of the splinting bar with four implants was simulated by rigid contact when the possibility of frictionless slipping between the implant head surfaces and the sockets (fastening holes of the splinting bar) was excluded. These conditions reflect the tight fit of the dental structure components. The clamps for fastening the ROD to the splinting bar were excluded from this model. Instead, rigid connections were introduced as contact surfaces, attaching the ROD to the splinting bar. To consider such cases, the virtual fastener (joint) options were activated in the ‘ANSYS 19’ software package. These contact surfaces are represented by green areas in Figure 3. Initially, four arbitrary asymmetric positions of their attachment (retention zones) were selected. As a result, the space between the splinting bar and the ROD base in the specified areas is provided with a virtual rigid connection, limiting the orthogonal movement between the mating surfaces. At the same time, this allows for the unimpeded transfer of loads between the contacting components of the dental structure.

When assessing the structural integrity of the elements of the dental structure, a safety factor (*n*) was introduced. If σ is the performance parameter defined in engineering analysis as the equivalent von Mises stress in the studied area of the element of the dental structure, then the safety factor is defined as follows:

(1) $$n=\frac{<\sigma >}{\sigma_{\max}},$$

where <σ> is the critical (limit) stress for a material that disrupts the normal operation of a product, and σ_max_ is the greatest calculated value of the parameter under the given loading conditions. The structural integrity condition is written as *n* ≥ 1, as the permissible value of the safety margin; in the favorable case (under static loads), its value should be 2 or 3.

The minimum limit for spongy bones, which was determined experimentally depending on patient age [24,25], was taken as the critical value <σ> for the bone tissue of the skull’s facial part. The normal mineralization of bone tissue was considered to be ~13 MPa.

Polymethyl methacrylate (PMMA) was selected as the material of the ROD, as it is widely used for this purpose [26]. According to previously reported data [27], the ultimate tensile strength (UTS) of PMMA varies from 47 to 79 MPa. In this study, a minimum value of 47 MPa was used. The mechanical properties of the Ti-6Al-4V alloy were used for the implants and splinting bar [28,29], in particular, its UTS level of 930 MPa. The physical and mechanical properties of the materials used in the computer simulation are listed in Table 1.

**Figure 4:**
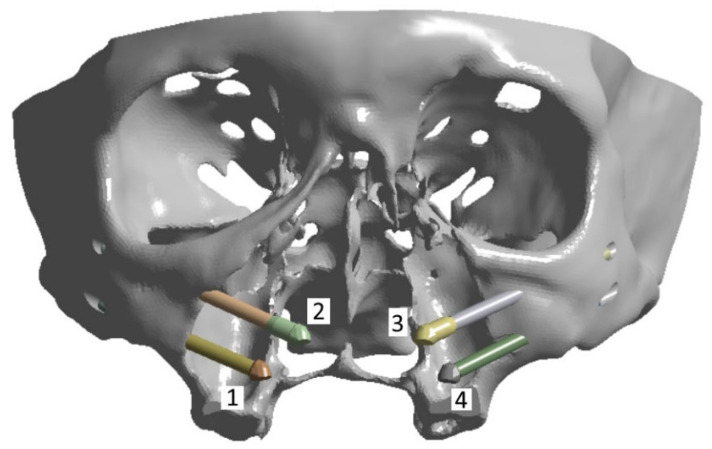
The implants numbering used in computer simulation: (**1**) and (**4**) ONC–55-37.5; (**2**) and (**3**) ZYGAN-47.5.

Four titanium implants of two types were considered in the model (Figure 4). The upper row (anterior) includes two ZYGAN-47.5 implants (No. 2 and No. 3). Their length was 47.5 mm, and the diameters of both the implant body and screw part in contact with the bone tissue were 3.4 mm [30]. The lower row (posterior) consisted of two ONC–55-37.5 implants (No. 1 and No. 4), with parameters of 37.5, 3.5, and 4.1 mm, respectively. The minimum depth of implant insertion into the zygomatic bone was set at 15 mm [30]. The implant numbering is illustrated in Figure 4. They were matched to those for the clamps on the ROD and bone tissue areas at the attachment regions for the corresponding implants.

In this work, the posterior implants #1 and #4 ONC–55-37.5 (37.5 mm long) were chosen to be shorter than the anterior implants #2 and #3 ZYGAN-47.5 (47.5 mm long) due to the geometry of the skull and the placement of the elements of the dental structure. During the surgery planning stage, the type of dental implant is selected by the oral surgeon, taking into account recommendations of the manufacturers. The attachment sites in the zygomatic bones are of particular importance [18]. In addition, due to the larger length of the anterior dental implants, it was decided to use ZYGAN-47.5 ones due to the presence of structural expansion in their apex. This made it possible to reduce or eliminate the stress concentration in the apex area due to the bending moment induced by the length of the implant.

Some researchers have analyzed the effects of masticatory muscles on the attachment regions of the zygomatic bones [16,31]. In this study, their action on the cranial bones was not considered since this boundary condition complicates the obtained results due to the impossibility of separating these levels from the overall values [32].

## 3. The Results of Computer Simulation

### 3.1. Evaluation of SSSs of the Dental Structure During Insertion and Removal of the ROD

For the ROD, both insertion and removal processes were simulated assuming that four clamps are serially snapped (with a force of 20 N/~1.8 kg on each) and unsnapped, respectively (to the right from the retention zone, i.e., No. 1–No. 4, in accordance with the numbers shown in Figure 3c). In Supplementary File S1, Table S1 presents the calculated data, where the first column indicates the loading location by the retention zones (from No. 1 to No. 4), followed by the maximum equivalent stresses in the corresponding components of the dental structure.

Figure S16 shows histograms plotted using the data presented in Table S1. These reflect the differences in equivalent stresses in the studied components of the dental structure for the retention zones under four studied loading conditions. The distribution of the maximum equivalent stresses in the splinting bar is uneven, with stress raisers in retention zones No. 2 and No. 3 during insertion/removal of the ROD using the clamps No. 2 and No. 3 (Figure S16a). In the implants, the maximum stresses are observed in the retention zones adjacent to the one used for fixing, while the others do not experience significant loads (Figure S16b).

**Figure 5:**
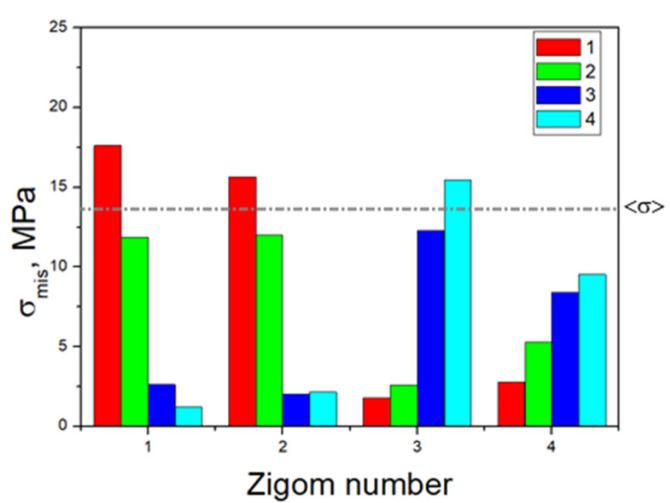
Histogram of the distribution of the maximum equivalent stresses (MPa) in the zygomatic bone during the serial insertion/removal of the ROD.

The minimally loaded areas of the zygomatic bones, which do not lead to a critical state, are adjacent to implant No. 4 in all cases. In Figure 5, the dashed line indicates the <σ> ultimate stress of 13 MPa for the zygomatic bones (Table 1); the equivalent stresses do not reach this level for the other two elements.

By applying the critical stresses of the materials (Table 1) in the strength analysis, the following minimum safety factors (*n*) are determined:

– 10.6 (*n* > 1) in the splinting bar, the junction area of retention zone No. 3, when fixing clamp No. 2;
– 31 (*n* > 1) in the second implant when fixing clamp No. 1;
– 0.72 (*n* < 1) in the zygomatic bones, the area of attachment of implant No. 1, when fixing the removable overdenture using clamp No. 1;
– 0.86 (*n* < 1) in the zygomatic bones, the areas of attachment of implants No.2 and No.3, when fixing clamps No. 1 and No. 4, respectively.

**Figure 6:**
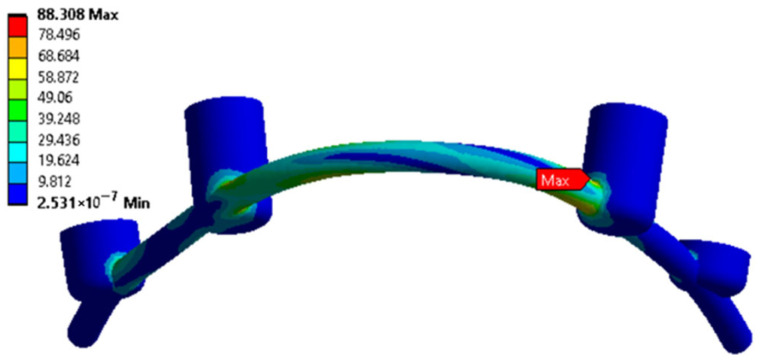
An example of equivalent stress distributions (MPa) in the splinting bar upon fixing the clamps in the retention zones; loading case No. 2 (according to Figure 3c).

**Figure 7:**
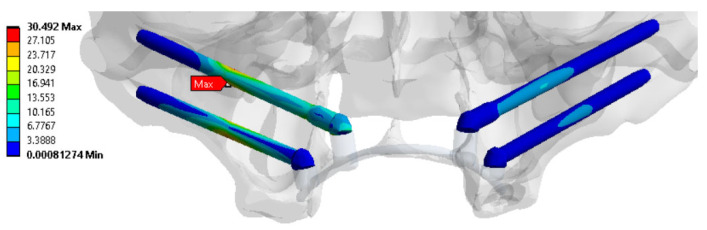
An example of equivalent stress distributions (MPa) in the implants upon fixing the clamps in the retention zones, loading case No. 1 (according to Figure 3c).

**Figure 8:**
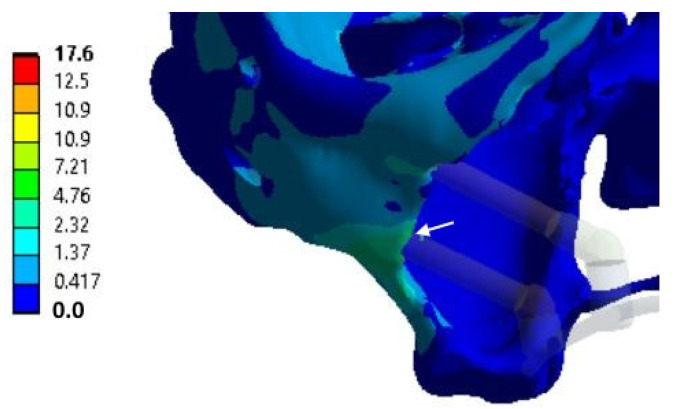
An example of equivalent stress distributions (MPa) in the zygomatic bones upon fixing the clamps in the retention zones; loading case No. 1 (according to Figure 3c); the white arrows indicates the region of maximum values.

For the three cases with *n* < 1, the specified loading conditions are critical for the bone tissue. The red color in Table S1 indicates that the calculated stresses exceed the <σ> level (according to the material properties presented in Table 1). In all other cases, *n* > 1, reflecting acceptable operating loads within the preset limits. To fix the clamps, some examples of the distributions of equivalent von Mises stresses for the splinting bar, implants, and zygomatic bones are shown in Figure 6, Figure 7 and Figure 8, respectively, where arrows indicate the areas of the maximum <σ> values. An additional part of the illustrations with the calculation results is reported in the Supplementary File S2, Figures S1–S4.

The data are provided for only one considered case, as the element is first mentioned in the text. All other illustrative materials concerning the same elements under other loading conditions are provided in Supplementary File S2.

Based on the above, the critical (threshold) stresses exceeded only in the zygomatic bones when loading clamps No. 1 and No. 4, i.e., along the edges of the splinting bar. However, this phenomenon is observed in three of the 16 positions in the four considered cases of fixing the ROD to the splinting bar with clamps (Figure 5).

Under the conditions applied in computer simulation, it can be assumed that insertion and removal of the ROD do not give rise to fractures of the zygomatic bones in the regions of attachment of the implants. Therefore, the critical equivalent stresses in the zygomatic bones do not depend on the loading direction, namely, insertion/removal of the ROD. This is suggested to be due to the effective consolidating role of the splinting bar that redistributes the applied loads when its type (tension or compression) does not change the magnitude of stresses (in the regions of attachment of the implants to the zygomatic bones).

### 3.2. Determination of SSSs in the Components of the Dental Structure Under Static Loads (Mastication on Molars and Premolars)

**Figure 9:**
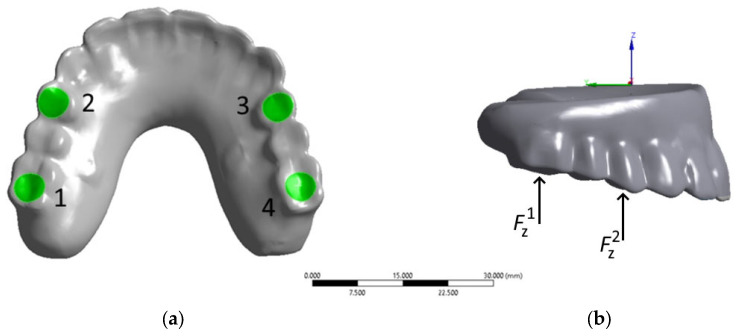
Scheme of static loading on the numbered areas of the posterior artificial teeth under mastication: (**a**) Bottom view with loading areas; (**b**) Right view with coordinate system axis.

The next considered cases are characterized by loading on the posterior blocks of teeth (molars and premolars) under mastication. This process was simulated as a serial application of a load of 50 N to the ROD in positions No. 1–4 on the contact surfaces of the teeth, as shown in Figure 9a (in the normal direction to the occlusion surfaces along the Z-axis, designated by the local coordinate system in Figure 9b).

Note that each implant interacts with the ROD at individual attachment points through the splinting bar, to which it is attached using four clamps in the retention zones. Thus, uneven redistributions of the applied loads to the artificial teeth of the ROD may occur. This fact is confirmed by the results of the computer simulation presented below, which assesses the redistribution of reactive forces on the implants for each loading option.

The calculated redistributions of reactive forces on the bases of the zygomas from the impact on the posterior blocks of the artificial teeth of the ROD in the area of contact of the heads of the zygomas and the splinting bar are presented in Table 2. The numbers of the zygoms from one to four horizontally correspond to those of the implants, as shown in Figure 4. The number of areas of application of the loads vertically is consistent with that of the loaded artificial teeth, as shown in Figure 9a.

Figure S17 shows a histogram plotted using the data presented in Table 2, which reflects the uneven redistributions of reactive forces in the implants caused by application of loads to different areas of the ROD under mastication. The quantitative analysis of reactive forces in the implants has proven the previously suggested hypothesis of their uneven redistributions under mastication. For each applied load, the total values are similar to the applied levels (within small errors). This is because of the parallelism of the load vectors to the tooth occlusion zone and the reactive forces arising in the regions of attachment to the zygoma; otherwise, this condition is not met. When loading on occlusion areas No. 2, No. 3, and No. 4, the direction of the reactive forces on the ROD changes its sign to the opposite in implants No. 4, No. 1, and No. 2, respectively.

The results of the qualitative assessment of the stress distributions indicated that the implants perceived bending moments in all cases. However, with the specified features of the redistributions of reactive forces, one of the four zygomas experiences a bend in the opposite direction; therefore, the resulting axial load contributes to conditions for pulling or tearing out the implants from the zygomatic bones. Under mastication, the only implant, No. 3, is deformed in one direction, while the others change the sign of the resulting axial force depending on the load application site.

**Figure 10:**
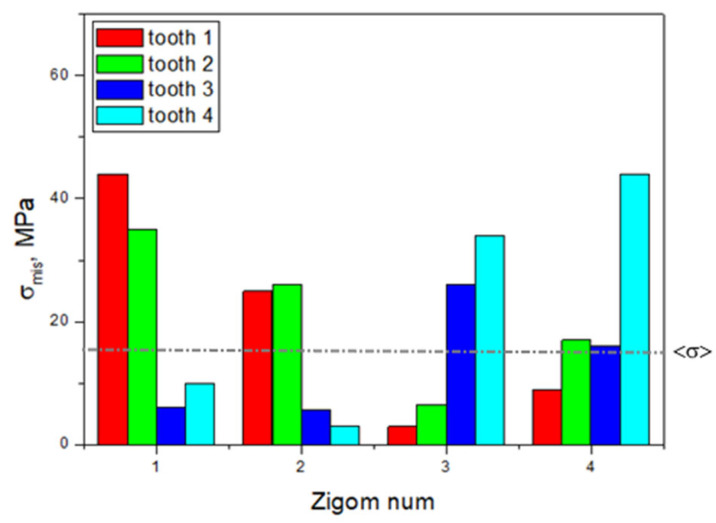
The histogram, plotted using the data presented in Table S2, reflects the distribution of the maximum equivalent stresses in the zygomatic bones during mastication.

The calculated equivalent stresses for the load redistributions under mastication are listed in Table S2. Unlike the previous case, their values in the retention zones of the ROD are also included. Figure S18 shows histograms plotted using the data presented in Table S2, which reflect the uneven distributions and variations in the elements of the dental structure under the influence of the loads on the posterior blocks of the artificial teeth. In Figure 10, the dashed line indicates the <σ> ultimate stress from Table 1; for the other three elements, the equivalent stresses do not reach the critical level.

By applying the critical stresses of the materials (Table 1) in the strength analysis, the following minimum safety factors (*n*) were determined:

– 2.4 (*n* > 1) in retention zone No. 4 of the ROD when loading tooth No. 4 (molar);
– 3.9 (*n* > 1) in retention zone No. 4 of the splinting bar when loading tooth No. 4 (molar);
– 14.8 (*n* > 1) in implant No. 4 when loaded on tooth No. 4 (molar).
– 0.3 and 0.52 (*n* < 1) in the implant attachment areas No. 1 and No. 2 of the zygomatic bones when loading tooth No. 1 (molar);
– 0.37 and 0.5 (*n* < 1) in the implant attachment areas No. 1 and No. 2 of the zygomatic bones when loading tooth No. 2 (premolar);
– 0.5 and 0.81 (*n* < 1) in the implant attachment areas No. 3 and No. 4 of the zygomatic bones when loading tooth No. 3 (premolar);
– 0.65 (*n* < 1) in the implant attachment areas No. 3 and No. 4 of the zygomatic bones when loading tooth No. 4 (molar).

When *n* < 1, the specified loading conditions are critical for the stresses arising in the zygomatic bones.

**Figure 11:**
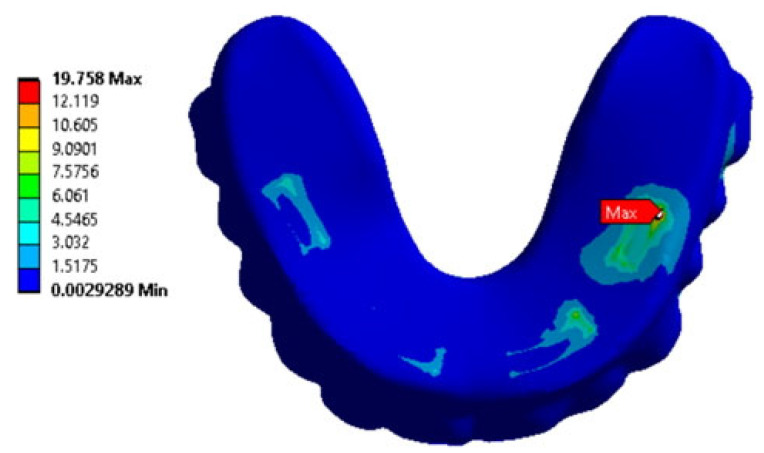
An example of equivalent stress distributions (MPa) in the retention zones of the ROD (under mastication) in loading case No. 4 (according to Figure 9a).

Figure 11 shows equivalent stress distributions when they are localized in the retention zones of the ROD (the clamps according to Figure 3b) under mastication for all four loading conditions.

Some examples of the distributions of the equivalent von Mises stresses in the splinting bar, implants, and zygomatic bones under mastication are shown in Figures S5–S7. The arrows indicate the regions of their maximum values.

Therefore, it was shown that mastication with localized normal loading on individual posterior artificial teeth in the ROD with a force of 50 N can cause critical stresses of about 16–44 MPa in each of the four areas of attachment of the implants in the zygomatic bones. When loading teeth No. 1 and No. 2, the maximum stresses were observed at the area of attachment of implant No. 1. They are located near the upper and lower implants No. 3 and No. 4 for the same teeth in both cases.

The authors suggested that this is due to the variations in both implant attachment areas and lever lengths from them to the splinting bar, contributing to the bending moments.

### 3.3. Calculation of SSSs in the Components of the Dental Structure Under Biting by the Incisors

**Figure 12:**
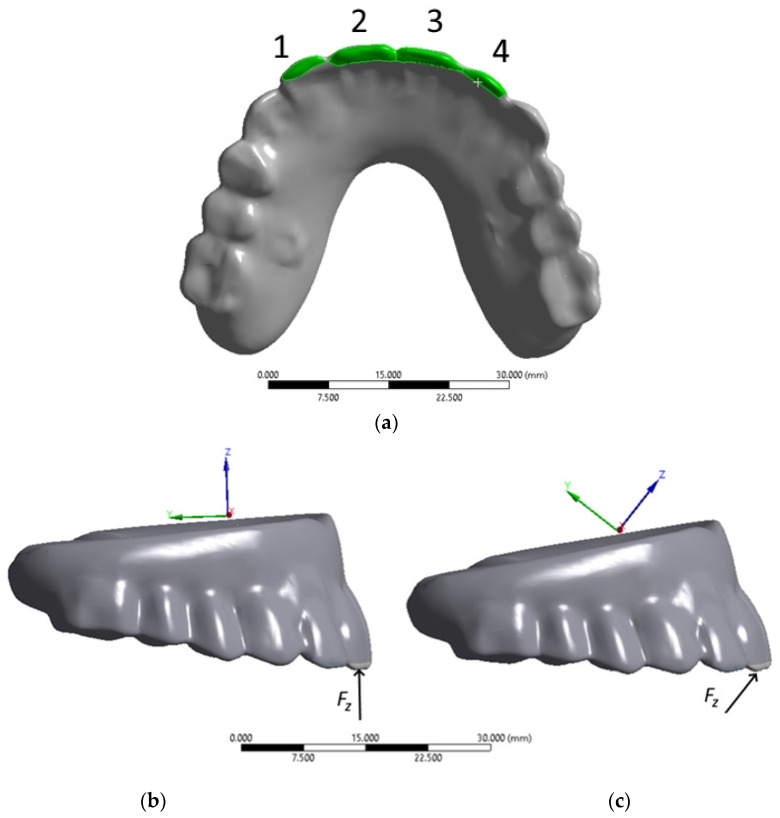
Load application areas on the artificial incisors during anterior biting: (**a**) Bottom view with loading areas; (**b**,**c**) Right view with coordinate system axes for loading angles of 90° and 45°, respectively.

In a computer simulation of the biting process, a load of 50 N was serially applied to the artificial incisors of the ROD (Figure 12a):

- in the positions Nos. 1–4 (Figure 12b) at angles of 90°
- and 45° according to the local coordinate system shown in Figure 12c.

By analogy with the above cases, the redistributions of reactive forces in the implants upon loading on the artificial incisors (Figure 12) were assessed at the contacts of the implant heads and splinting bar. The obtained results are presented in Table 3 and Table 4, respectively, and in Figure S19 as histograms. The numbers from one to four horizontally correspond to those of the implants, while vertically they reflect the loaded areas of the ROD, Figure 12a.

By performing a comparative analysis of the histograms presented in Figure S19, it was concluded that loading at an angle of 45° increased the reactive forces in implants No. 1 and No. 4 by almost two times than those at an angle of 90°. In general, the patterns revealed were qualitatively similar. At the same time, the greatest reactive forces were developed in the implants No. 2 and No. 3 of the upper row, close to the load application area on the ROD.

**Figure 13:**
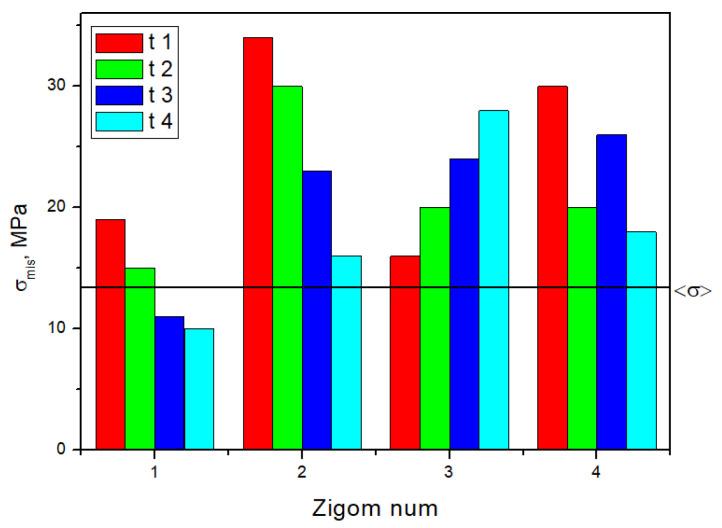
The histogram plotted using the data presented in Table S3 reflects the distributions of the maximum equivalent stresses in the zygomatic bones during 90° anterior biting.

The maximum equivalent stresses in the components of the dental structure upon loading on the artificial incisors at an angle of 90° are given in Table S3, Figure S20 and Figure 13 as histograms. The equivalent stress distributions in the retention zones of the ROD are even in all studied cases. In the splinting bar, the implants and zygomatic bones showed peaks in retention zones No. 2 and No. 3, upper implants No. 2 and No. 3 related to the anterior part of the dental structure, and zygomatic bones in positions No. 2 and No. 3.

By applying the critical stresses of the materials (Table 1) in the strength analysis, the following minimum safety factors (*n*) were determined:

– 6.0 (*n* > 1) in retention zone No. 3 of the ROD when loading tooth No. 4;
– 3.7 (*n* > 1) in retention zone No. 3 of the splinting bar when loading tooth No. 1;
– 16.3 (*n* > 1) in implants No. 2 and No. 3 when loading teeth No. 1 and No. 4;
– 0.38 and 0.52 (*n* < 1) in the implant attachment area No. 2 of the zygomatic bones when loading tooth No. 1;
– 0.86, 0.43, and 0.65 (*n* < 1) in implant attachment areas No. 1–4 of the zygomatic bones when loading tooth No. 2;
– 0.56, 0.54, and 0.5 (*n* < 1) in the implant attachment areas No. 2–4 of the zygomatic bones when loading tooth No. 3;
– 0.81, 0.46, and 0.72 (*n* < 1) in the implant attachment areas No. 2–4 of the zygomatic bones when loading tooth No. 4;

When *n* < 1, the specified loading conditions were critical for the stresses in the zygomatic bones.

**Figure 14:**
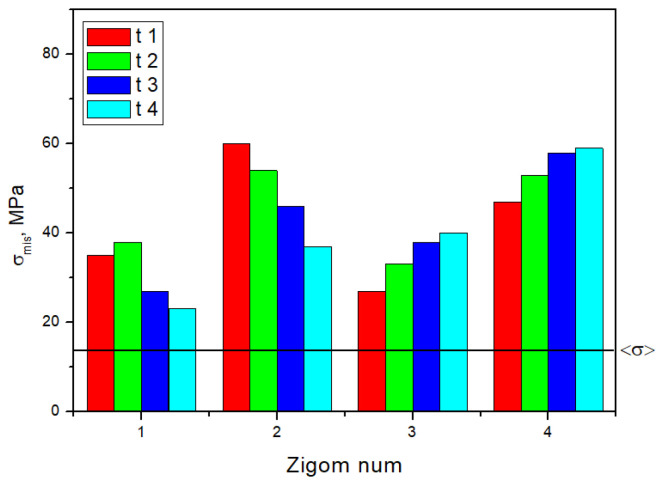
The histograms plotted using the data presented in Table S3 reflect the distributions of the maximum equivalent stresses in the zygomatic bones during 45° anterior biting.

The maximum equivalent stresses in the components of the dental structure upon loading the artificial incisors at an angle of 45° are shown in Table S4 and Figure S21, and for the zygomatic bones in Figure 14 as histograms.

By applying the critical stresses of the materials (Table 1) in the strength analysis, the following minimum safety factors (*n*) were determined:

– 3.6 (*n* > 1) in retention zone No. 1 of the ROD when loading on tooth No. 4;
– 3.0 (*n* > 1) in retention zone No. 3 of the splinting bar when loading tooth No. 1;
– 16.3 (*n* > 1) in implant No. 4 when loaded on tooth No. 4;
– 0.22 in the implant attachment area No. 2 of the zygomatic bone when loading tooth No. 1.

The equivalent stresses exceeded their acceptable level in all four implants for all studied cases, with the safety factors changing from 0.22 to 0.57 (*n* < 1). When *n* < 1, the specified loading conditions were critical for the stresses developing in the zygomatic bones.

For the splinting bar, implants, and zygomatic bones, some examples of the distributions of equivalent von Mises stresses under biting are shown in Figures S8–S15, respectively, where arrows indicate the areas of their maximum values.

Thus, biting-induced local loading of 50 N on individual artificial incisors of the ROD can cause critical stresses in the zygomatic bones of 15–34 MPa (at an angle of 90°) and 23–60 MPa (at an angle of 45°) for each of the implant attachment areas. When loading teeth No. 1 and No. 2, the maximum stresses were observed at the implant attachment area No. 2 in all the studied cases. However, they developed at implant attachment areas No. 4 and No. 3 when loading teeth No. 3 and No. 4.

## 4. Discussion

In most cases of partial or full maxillectomy, the above-mentioned zygomatic implants are successfully used with their attachment by a two-point support scheme [18,33]. At the same time, a combined scheme of their installation is recommended [1,21,33]: (i) a pair of zygomatic implants is fixed in the zygomatic bone on the affected side, and (ii) two to three conventional implants are attached to the alveolar bone on the unaffected side. The results of FEA and subsequent clinical studies have shown that installing two long implants on the affected side of the zygomatic bone instead of only one is preferable. This is because the use of two implants allows for more effective load redistribution [32].

The calculation results on the pairwise attachment of zygomatic implants without combining them with short dental implants were considered in [1,15,17,21], where the main load from mastication was transferred to the zygomatic bones. Moreover, Ujigawa, K. [15] considered a case in which zygomatic implants were installed according to a single–support scheme, with only one end fixed in the bone. The validity of this installation method was confirmed by analytical calculations conducted in [18]; however, the used attachment scheme multiplies the stresses in the implant as well as in the adjacent bone compared to the two-point support scheme. The above-cited papers [15,17] reported that splinting with a stiff arch is similar to the principle of a maxillary prosthesis. This makes it possible to better redistribute the loads over the areas of all implant attachments.

In this paper, we continue to theoretically study the problem of using zygomatic implants under the treatment of the maxillectomy [33]. To the best of our knowledge, the literature lacks descriptions of clinical cases with successful treatment results using this scheme. It is suggested that an FEA stage should be added for efficient surgery planning. In the development of the problem considered in [15], the current study deals with a model advanced by adding a titanium splinting bar and PMMA maxillary prosthesis.

Using the CT-DICOM data of a patient after total maxillectomy, the applied computer simulation allowed the visualization and analysis of the distributions of stresses in the (i) zygomatic bones, (ii) titanium implants, (iii) splinting bar, and (iv) polymer ROD. The developed 3D model of the dental structure provided detailed information on the anatomical features of the regions of interest, such as maxillary sinuses and craniofacial bones. It should be noted that the CT data of a real patient assumed certain variations in the contact areas of the implants with the zygomatic. For this reason, the stresses arising in these areas are different. In addition, implants of two standard sizes were used, which should also vary the stresses arising in the zygomatic bones.

**Figure 15:**
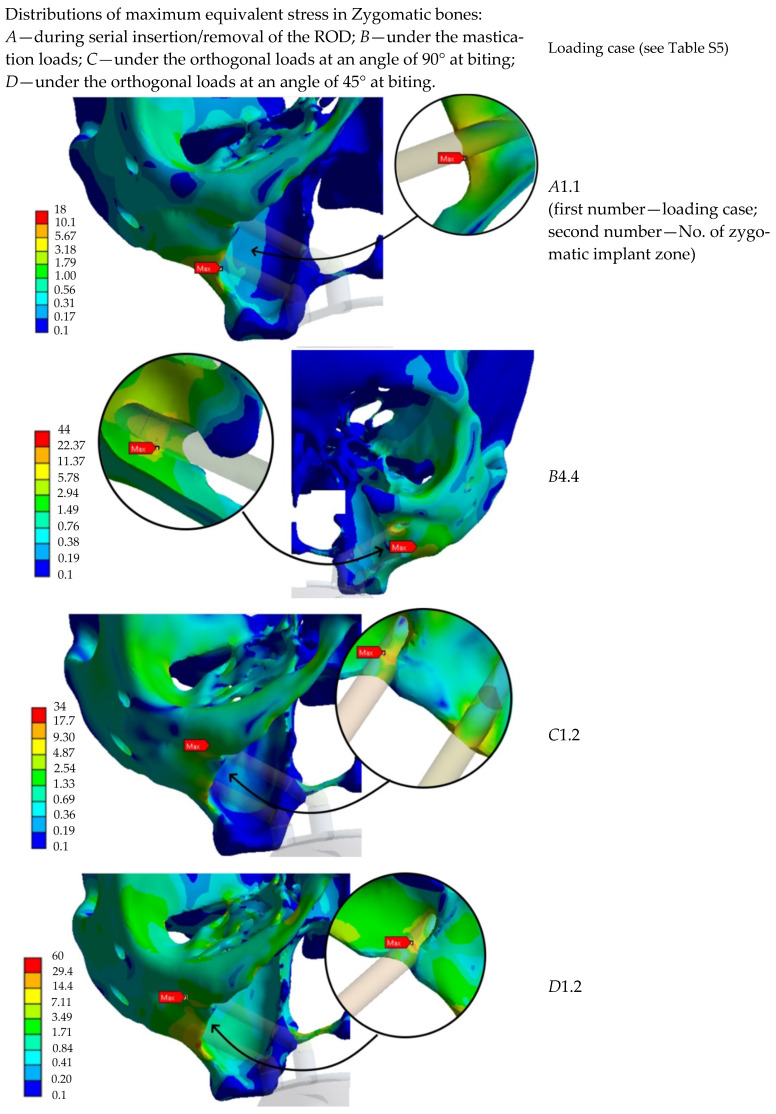
Characteristic stress maps for the results in Table S5, illustrating the distribution of stress intensity values (MPa) in the zygomatic bones under the most critical loading conditions.

Table S5 summarizes the results of the comparison of the maximum von Mises stress values across all anatomical regions and loading conditions. The maps of stress intensity values (MPa) under the most critical loading conditions are shown in Figure 15. This helps generalize the key calculation results.

The implants were installed at angles to the occlusal plane; therefore, the vertical forces led to the development of lateral forces in all the studied cases (under both mastication and biting, as well as during insertion and removal of the detachable overdenture). The latter causes bending moments. However, the shunting action of the splinting bar increases the stiffness of the dental structure. At the same time, the occlusal loads were unevenly redistributed among the four implants.

The first original result that requires discussion is the closeness of the stress values that arose during both the insertion and removal of the ROD (i.e., under the influence of both compressive and tensile stresses). The equivalent von Mises stress is an invariant of the stress tensor and is a scalar quantity. Thus, the compressive or tensile components of the strain tensor included in the expression for calculating the equivalent stress are the same in absolute values during both the insertion and removal of the ROD. This suggests that a force of similar magnitude but different in sign (direction) acts on the same sections of the dental structure in both cases. Therefore, the components of the principal stress tensor have different signs, but they are equal in absolute value. As a result, the equivalent stresses are the same in both cases since they are equal to the root of the squared differences of the principal stress tensor components. The splinting bar, attached to the base at one end, gives the same response, estimated by the equivalent stresses, when similar forces of different signs act on its unconfined end. Thus, despite the complex shape of the elements of the dental structure, this is true for them, similar to the case of fixing the splinting bar to a simple base.

The next important result is the difference in the critical stresses when loading the posterior and anterior teeth. Under mastication, half of the equivalent stresses of the 16 studied do not exceed the threshold level. In the 45° biting loading, all values are higher, while only two are lower at 90°. In general, the obtained results are not unexpected, as loading on the anterior teeth leads to the development of tilting forces, additionally affecting both the implants and zygomatic bones.

Finally, the difference in the stress levels was revealed upon axial and off-axis loading on the anterior teeth (under biting), including both the magnitude and the location of the critical stresses. At the same time, the stress values are higher for all components of the dental structure for the 45° biting loading than for the 90°. The reason for this is explained in the previous section. According to the obtained results, the design of the dental structure should be considered acceptable, taking into account the use of the splinting bar as a method of fixing the ROD. As a result, the stiffness of the dental structure was improved, and the stresses were effectively redistributed between the implants consolidated by the splinting bar.

The authors suggest that the following aspects might be treated as practical recommendations based on the results obtained in this study:

- The cross-section of a splinting bar is usually minimized in order to maintain the compact dimensions of the RODs. This reduces the stiffness of the bar, which can cause its bending and fatigue failure as well. The estimation of stress obtained during computational experiments makes it possible to modify the design (shape) of the bar with a view to its reinforcement.
- Analysis of the simulation results made it possible to identify the regions of maximum stress concentration in the splinting bar. Among them are sites of abrupt geometric transitions that are adjacent to the attachment heads. It is recommended to redistribute the stresses by excluding/minimizing sharp stress risers.
- The use of a non-circular cross-section of the splinting bar in combination with its topological optimization would allow the stress concentrations to be leveled.
- Regarding the design of the dental system, it is recommended to change the type or length of implants as well as their number, since the overdenture can be attached to 2–6 zygomatic implants.
- In addition, based on the calculation results, patients can be visually instructed on the cautious application of such systems, and it is expected that high anterior biting forces can be sustained.

Despite the large number of simulation results presented above, this study is not free from limitations. Among them are:

- the assumptions of linear material properties, the use of which simplifies the obtained results. This is acceptable as a rough approximation, but the non-linear response of the system should be analyzed in further studies on the topic.
- The model of the dental system was simplified by excluding soft tissue or muscle forces.
- Static loading conditions are delicate and do not characterize the deformation behavior of the dental system under analysis well.
- Cyclic loading (fatigue) is the most critical factor in the long-term functioning of the dental system under analysis [34]. However, this was not the focus of this paper.

In order to illustrate the activity of the authors in the area of rehabilitation of cancer patients, a brief description of a clinical case with the use of dental implants and a splinting bar is provided below.

**Figure 16:**
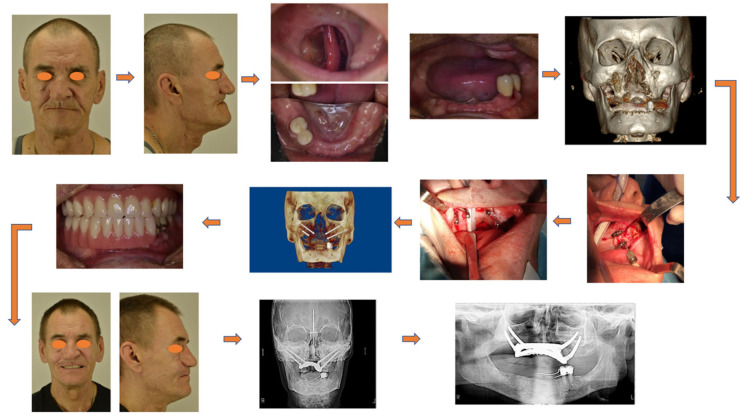
Clinical case of cancer patient rehabilitation with the use of dental implants and a spinting bar.

The 66-year-old patient was hospitalized with a diagnosis of “a combined defect of the middle zone of the face on the right side, including (i) the maxillary, (ii) hard palate and vestibule of the oral cavity, (iii) middle and lower parts of the maxillary sinus, and (iv) zygomatic bone. The reason was extensive tissue resection due to cancer of the mucous membrane of the maxillary bone on the right side”. Clinical examination of the face revealed a clear asymmetry, which consisted of displacement of soft tissues in the eye socket and buccal region, as well as the wings of the nose on the right and left sides (Figure 16).

With the help of preoperative computer-aided planning, the option of installing oncological zygomatic implants with a polished part of the implant body in the zygomatic bone and standard zygomatic implants from the opposite side was modeled.

During the surgery, a silicone impression of the position of the implants was obtained. Subsequently, a metal bar with screw fixation of the implants and an ROD was fabricated. The dental prosthesis was fixed on the splinting bar installed on the dental implants within 72 h after the operation.

The satisfactory level of retention of the implants (torque of 75 N) in the zygomatic bone made it possible to immediately load them using the ROD. Occlusal contacts with the artificial denture row in the mandible were verified. At least two pairs of teeth were in contact with the antagonists on each side of the RODs. Articulation of the mandible was unhindered.

Three months later, a control radiographic inspection was performed in direct projection (TRG) and orthopantomogram (OPTG) in order to confirm the tight contact between the ROD and the bar splinting the dental implants. By doing so, the successful rehabilitation of the patient was achieved.

**Figure 17:**
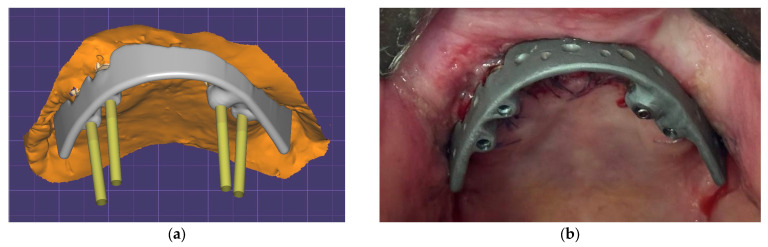
3D-digital model of a splinting bar (**a**) and photograph of a real dental structure being fixed at the implants (**b**).

Before reaching a conclusion, the following aspects must be outlined. Currently, practicing dentists and cranio-maxillofacial surgeons have been proposing and using a large number of designs for fixing a ROD with the help of a splinting bar. A variant similar to that described in the literature [21] was considered. In the authors’ own practice of maxillofacial prosthetics, other variants of dental structures are used, as shown in Figure 17. While taking into account the results obtained in this paper, the authors plan to conduct a finite element parametric study focused on determining optimal design solutions for such a splinting bar structure, as well as acceptable levels of functional loads.

## 5. Conclusions

The results of the computer simulation enabled us to draw the following conclusions.

1. The serial insertion and removal of the detachable overdenture caused identical stresses in the elements of the dental structure. In both cases, the equivalent stresses are equal and do not exceed the critical (fracture) levels for either the removable overdenture or the splinting bar, and especially for the implants. Given the accepted level of critical stress in the zygomatic bones of about 13 MPa, their values may be exceeded during both processes.
2. In the removable overdenture, the equivalent stresses do not exceed the critical levels upon serial loading of 50 N on the posterior teeth (both molars and premolars) under mastication in all studied cases. Taking into account the linear dependence of the applied load and the stresses in the removable overdenture, it can be stated that its integrity could be maintained till the load of 118 N (or the generally accepted typical value of 100 N). In the 90° anterior bite, the equivalent stresses are below the critical level in all the studied cases; therefore, the acceptable value increases up to 213 N, but it is only 63 N at a loading angle of 45°.
3. For all the loading cases studied, the equivalent stresses in the zygomatic bones exceeded the critical level of 13 MPa. Thus, given the specific condition of the patient’s bone tissue, whose CT data were used in the computer simulation, immediate rehabilitation using zygomatic implants (with the studied splinting bar) is not recommended. This is due to the potential loosening of the bone tissue and loss of implant retention.
4. Some calculations exceeded the zygomatic bone strain threshold. The possibilities for reducing the risk of dental bone failure are as follows: (i) changing the number, type, and size of the implants (zygomatic fixtures); (ii) changing the location of the implants’ fixation site; (iii) varying the thickness or hape of the splinting bar; (iv) reducing the retention of the clams; and (v) recommending that the patient handle (insert/remove) the overdenture in a more delicate way.

## Figures and Tables

**Table 1:** The physical and mechanical properties of the materials applied in computer simulation.

Model	*E*, GPa	υ	*G*, GPa	*K*, GPa	<σ>, MPa
Zygomatic bones	10	0.33	3.76	9.8	13
Removable overdenture	1.1	0.42	0.39	2.29	47
Titanium implants	96	0.36	35	114	930

where *E* is Young’s modulus, υ is Poisson’s ratio, *G* is shear modulus, *K* is bulk modulus, and <σ> is the critical stress level for the material.

**Table 2:** The redistributed reactive forces (*N*) in the implants under variable loads on the posterior teeth.

**Posterior Teeth**	**Loading Case**	**Implants**			
	**No.**	**1**	**2**	**3**	**4**
	**1**	−24.77	−17.47	−5.85	−0.95
	**2**	−12.31	−29.10	−17.28	9.64
	**3**	6.9	−13.88	−30.90	−11.16
	**4**	−7.33	3.10	−11.02	−33.78

**Table 3:** Redistributions of reactive forces (*N*) in the implants with serial loading on the artificial incisors at an angle of 90°.

**Incisors**	**Loading Case**	**Implants**			
	**No.**	**1**	**2**	**3**	**4**
	**1**	8.99	−41.07	−48.84	23.41
	**2**	14.89	−39.44	−55.90	22.63
	**3**	18.00	−34.98	−59.33	18.99
	**4**	18.11	−28.20	−57.05	11.44

**Table 4:** Redistributions of reactive forces (*N*) in the implants with serial loading on the artificial incisors at an angle of 45°.

**Incisors**	**Loading Case**	**Implants**			
	**No.**	**1**	**2**	**3**	**4**
	**1**	31.26	−52.26	−61.96	43.43
	**2**	36.87	−53.25	−66.60	43.44
	**3**	39.41	−51.10	−68.70	40.86
	**4**	39.60	−47.10	−66.85	34.81

## Supplementary Materials

The following supporting information can be downloaded at: https://www.mdpi.com/article/10.3390/dj13090393/s1, File S1: tables; File S2: figures. Table S1: The maximum equivalent stress distributions (MPa) in the elements of the dental structure during serial insertion/removal of the detachable overdenture; Table S2: The maximum equivalent stress distributions (MPa) in the components of the dental structure under the mastication loads; Table S3: The maximum equivalent stress distributions (MPa) in the components of the dental structure under the orthogonal loads at the angle of 90° at biting; Table S4: The maximum equivalent stress distributions (MPa) in the components of the dental structure under the orthogonal loads at the angle of 45° at biting; Table S5: The comparison of maximum von Mises stress values across all anatomical regions and loading conditions; Figure S1: The equivalent stress distributions (MPa) in the splinting bar upon fixing the clamps in the retention zones: (a) No. 1, (b) No. 2, (c) No. 3, (d) No. 4, respectively (according to Figure 3c). The letters (a–d) in the color legend indicate levels of maximum values of equivalent stresses corresponding to every loading case; Figure S2: The equivalent stress distributions (MPa) in the implants upon fixing the clamps in the retention zones: (a) No. 1, (b) No. 2, (c) No. 3, (d) No. 4, respectively (according to Figure 3c); Figure S3: The equivalent stress distributions (MPa) in the zygomatic bones upon fixing the clamps in the retention zones: (a) No. 1, (b) No. 2, (c) No. 3, (d) No. 4, respectively (according to Figure 3c); Figure S4: The equivalent stress distributions (MPa) in the retention zones of the removable overdenture at ‘mastication’: (a)–(d) loading on the tooth No. 1–4, respectively (according to Figure 9a). Figure S5: The equivalent stress distributions (MPa) in the splinting bar according to the results of computer simulation; the mastication loading; Figure S6: The equivalent stress distributions (MPa) in the implants according to the results of computer simulation; the mastication loading; Figure S7: The equivalent stress distributions (MPa) in the zygomatic bones according to the results of computer simulation of the mastication loading; Figure S8: The equivalent stress distributions (MPa) in the removable overdenture according to the results of computer simulation; the of 90° biting loading; Figure S9: The equivalent stress distributions (MPa) in the splinting bar according to the results of computer simulation; the 90° biting loading; Figure S10: The equivalent stress distributions (MPa) in the implants according to the results of computer simulation; the 90° biting loading Figure S11: The equivalent stress distributions (MPa) in the zygomatic bones according to the results of computer simulation; the 90° biting loading; Figure S12: The equivalent stress distributions (MPa) in the removable overdenture according to the results of computer simulation; the 45° biting loading; Figure S13: The equivalent stress distributions (MPa) in the splinting bar according to the results of computer simulation; the 45° biting loading; Figure S14: The equivalent stress distributions (MPa) in the implants according to the results of computer simulation; the 45° biting loading; Figure S15: The equivalent stress distributions (MPa) in the zygomatic bones according to the results of computer simulation; the 45° biting loading; Figure S16: The histograms of the distributions of the maximum equivalent stresses (MPa) in the elements of the dental structure during serial insertion/removal of the detachable overdenture; (a) splinting bar; (b) implant number; Figure S17: The histogram, plotted using the data presented in Table 2, characterizing the redistributions of reactive forces (N) in the bases [30] of the implants when applying the variable loads under mastication; Figure S18: The histograms, plotted using the data presented in Table S2, reflecting the distributions of the maximum equivalent stresses in the components of the dental structure under mastication: (a) removable overdenture; (b) splinting bar; (c) implants; Figure S19: The histograms of the redistributions of reactive forces (N) in the implants upon loading on the artificial incisors at the angles of 90° (a) and 45° (b); Figure S20: The histograms plotted using the data presented in Table S3, reflecting the distributions of the maximum equivalent stresses in the components of the dental structure at 90° anterior biting: (a) removable denture; (b) splinting bar; (c) implants; Figure S21: The histograms plotted using the data presented in Table S4, reflecting the distributions of the maximum equivalent stresses in the components of the dental structure at 45° anterior biting: (a) removable overdenture; (b) splinting bar; (c) implants.

## Abbreviations

The following abbreviations are used in this manuscript:

CAD	Computer-Aided Design
CT	Computed tomography
FEM	Finite element method
ONC	Oncology Implant
PMMA	Polymethyl methacrylate
ROD	Removable overdenture
SSS	Stress-strain state
UTS	Ultimate tensile strength
ZYGAN	Zygan Implant
