# Supplementary material for: Computer Simulation of the Mechanical Behavior of the ‘Zygomatic Bones–Implants–Splinting Bar–Removable Overdenture’ Dental Structure Under Operational Loads
Source: Dent J (Basel). 2025 Aug 28;13(9):393. doi: 10.3390/dj13090393 (PMC12469048; doi:10.3390/dj13090393)
Supplement: Supplementary file 1 [file dentistry-13-00393-s001.zip › File S1 - tables.pdf]

dentistry journal

Supplementary 1

Table S1. The maximum equivalent stress distributions (MPa) in the elements of the dental structure during serial insertion/removal of the detachable overdenture.

Clamp	No.	Splinting bar	Implants	Zygomatic bones
**1**	1	42	29	18
	2	38.4	30.5	15
	3	37.4	6.9	1.8
	4	5.6	5.6	2.8
**2**	1	44.7	19.6	12
	2	72.2	26.2	12.0
	3	88.3	27.9	2.6
	4	19.3	9.5	5.3
**3**	1	10.7	6.3	2.6
	2	68.4	9.4	2.0
	3	70.8	27.9	12.3
	4	53.9	20.2	8.4
**4**	1	5.2	3.7	1.2
	2	34.2	5.9	2.2
	3	35.0	28.3	15.5
	4	37.6	20.14	9.5

Table S2. The maximum equivalent stress distributions (MPa) in the components of the dental structure under the mastication loads.

Loaded tooth	No.	Removable overdenture	Splinting bar	Implants	Zygomatic bones
**1**	1	5.6	169	54	44
	2	3.5	113	50	25
	3	2.4	101	12	2.9
	4	3.6	32	12	8.9
**2**	1	7.9	125	45	35
	2	4.1	94	53	26
	3	4.0	163	16	6.5
	4	4.6	68	22	17
**3**	1	3.4	33	16	6.0
	2	3.9	111	15	5.6
	3	4.2	127	48	26
	4	9.6	133	31	16
**4**	1	5.0	24	13	10
	2	2.9	63	16	3.0
	3	7.1	235	55	34
	4	20	225	63	44

Table S3. The maximum equivalent stress distributions (MPa) in the components of the dental structure under the orthogonal loads at the angle of 90 ° at biting.

Loaded tooth	No.	Removable overdenture	Splinting bar	Implants	Zygomatic bones
**1**	1	6.8	90	32	19
	2	5.6	114	57	34
	3	6.4	252	36	16
	4	7.6	135	45	30
**2**	1	6.9	82	33	15
	2	5.9	141	54	30
	3	6.7	207	46	20
	4	6.9	136	48	20
**3**	1	7.5	78	34	11
	2	5.6	168	47	23
	3	6.8	159	53	24
	4	6.8	95	46	26
**4**	1	5.5	74	32	10
	2	5.3	179	37	16
	3	7.8	117	57	28
	4	7.6	101	37	18
**<σ>**	47		930	930	13

Table S4. The maximum equivalent stress distributions (MPa) in the components of the dental structure under the orthogonal loads at the angle of 45 ° at biting.

Loaded tooth	No.	Removable overdenture	Splinting bar	Implants	Zygomatic bones
**1**	1	8.9	252	87	35
	2	6.9	109	75	60
	3	8.7	313	65	27
	4	13.2	189	73	47
**2**	1	11.4	224	82	38
	2	6.1	159	72	54
	3	8.7	279	75	33
	4	11.2	234	81	53
**3**	1	13.4	154	73	27
	2	6.3	202	65	46
	3	8.4	217	85	38
	4	10.2	279	89	58
**4**	1	14.4	145	67	23
	2	5.9	198	57	37
	3	10.7	131	91	40
	4	11.2	304	92	59
**<σ>**	47		930	930	13

Table S5. – The comparison of maximum von Mises stress values across all anatomical regions and loading conditions.

Loading case	No.	A, MPa	B, MPa	C, MPa	D, MPa
1	1	18	44	19	35
	2	15	25	34	60
	3	1.8	2.9	16	27
	4	2.8	8.9	30	47
2	1	12	35	15	38
	2	12.0	26	30	54
	3	2.6	6.5	20	33
	4	5.3	17	20	53
3	1	2.6	6.0	11	27
	2	2.0	5.6	23	46
	3	12.3	26	24	38
	4	8.4	16	26	58
4	1	1.2	10	10	23
	2	2.2	3.0	16	37
	3	15.5	34	28	40
	4	9.5	44	18	59

Loading case – numbering of loading regions for different studied cases;

No. – the number of zygomatic bone region, to which an implant is attached (Figure 4);

A – maximum equivalent stress distributions in Zygomatic bones during sequential insertion/removal of the detachable overdenture (Loading cases in Figure 9);

B – maximum equivalent stress distributions in Zygomatic bones under the mastication loads (Loading cases in Figure 3, c);

C – maximum equivalent stress distributions in Zygomatic bones under the orthogonal loads at the angle of 90° at biting (Loading cases in Figure 12, b);

D –maximum equivalent stress distributions (MPa) in Zygomatic bones under the orthogonal loads at the angle of 45° at biting (Loading cases in Figure 12, c).
