# Supplementary material for: Computer Simulation of the Mechanical Behavior of the ‘Zygomatic Bones–Implants–Splinting Bar–Removable Overdenture’ Dental Structure Under Operational Loads
Source: Dent J (Basel). 2025 Aug 28;13(9):393. doi: 10.3390/dj13090393 (PMC12469048; doi:10.3390/dj13090393)
Supplement: Supplementary file 1 [file dentistry-13-00393-s001.zip › File S2 - figures.pdf]

dentistry journal

MDPI

Supplementary 2

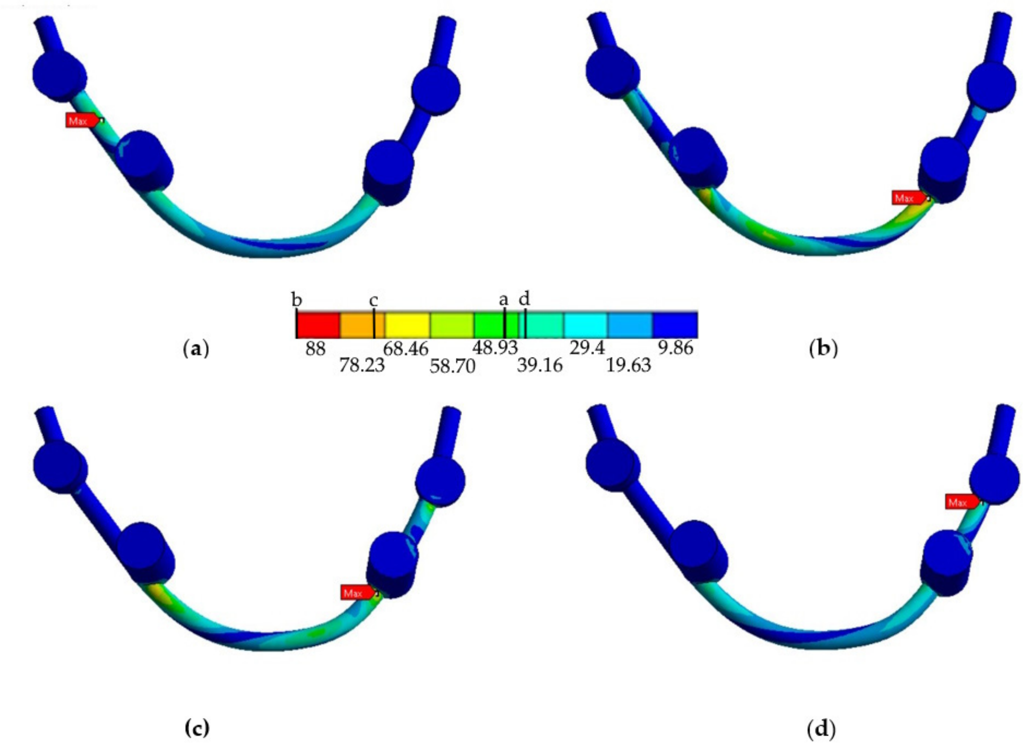

Figure S1 The equivalent stress distributions (MPa) in the splinting bar upon fixing the clamps in the retention zones: (**a**) No. 1, (**b**) No. 2, (**c**) No. 3, (**d**) No. 4, respectively (according to Figure 3, **c**). The letters (a-d) in the color legend indicate levels of maximum values of equivalent stresses corresponding to every loading case.

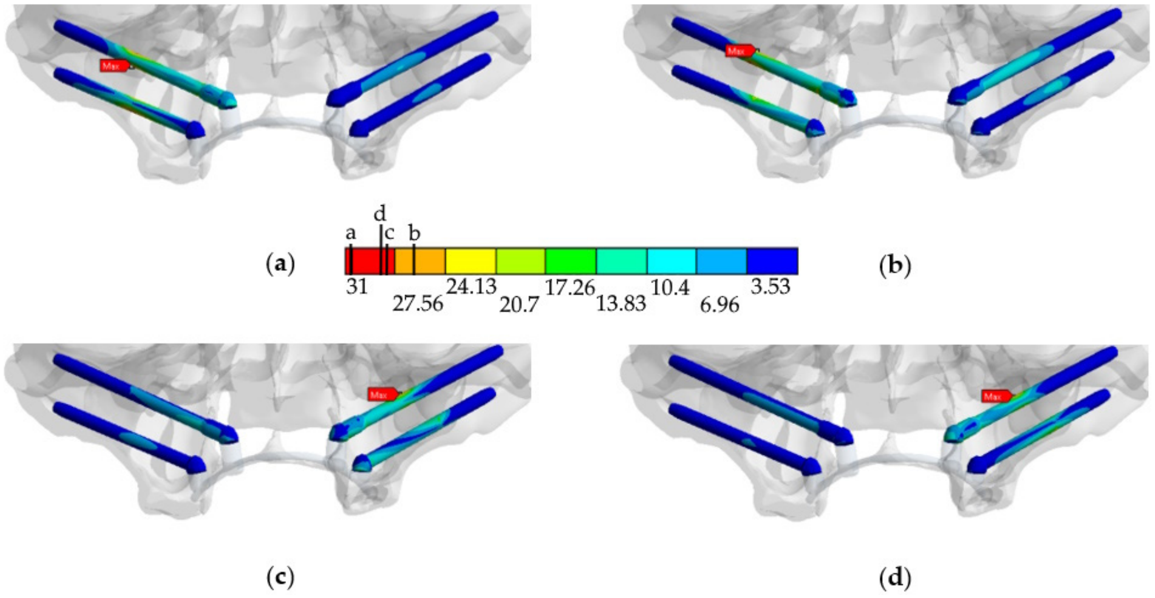

Figure S2. The equivalent stress distributions (MPa) in the implants upon fixing the clamps in the retention zones: (**a**) No. 1, (**b**) No. 2, (**c**) No. 3, (**d**) No. 4, respectively (according to Figure 3, **c**).

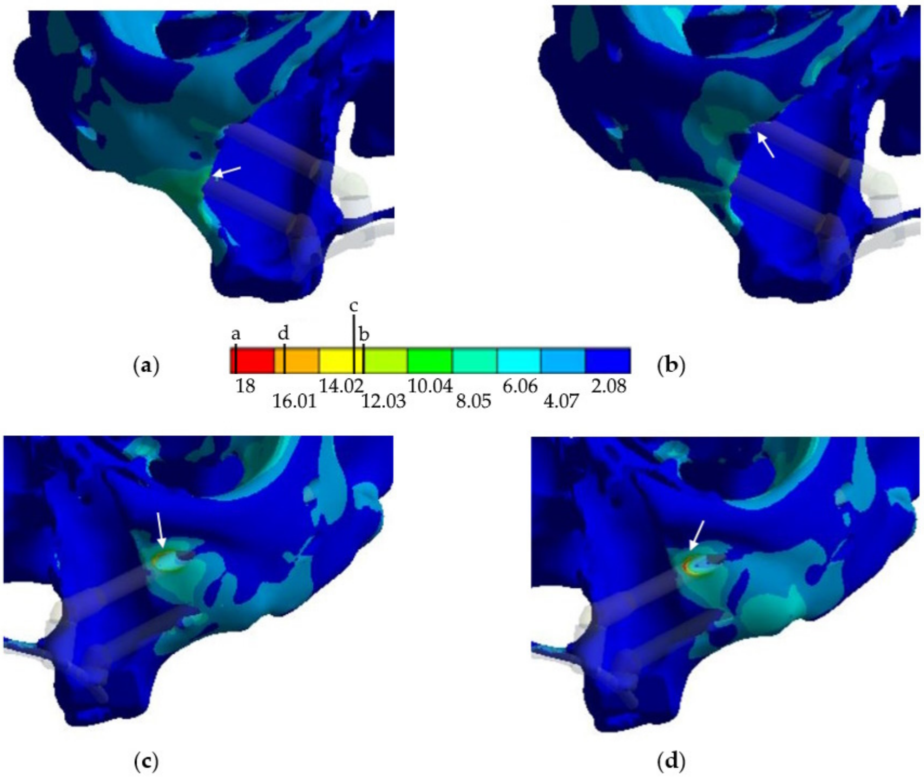

Figure S3 The equivalent stress distributions (MPa) in the zygomatic bones upon fixing the clamps in the retention zones: (**a**) No. 1, (**b**) No. 2, (**c**) No. 3, (**d**) No. 4, respectively (according to Figure 3,**c**).

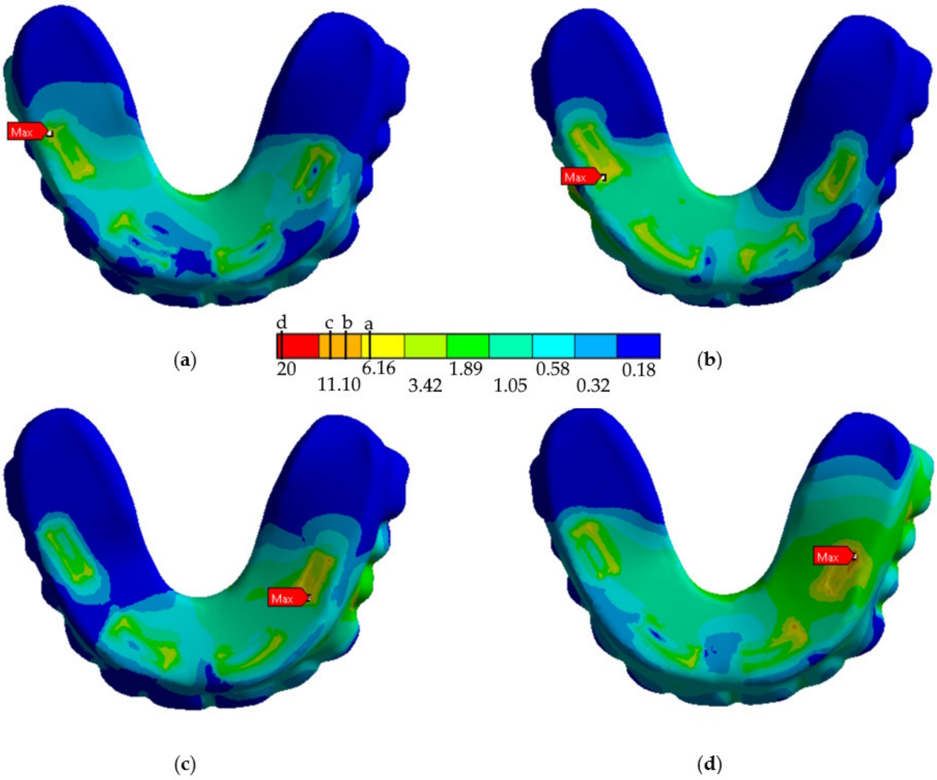

Figure S4 The equivalent stress distributions (MPa) in the retention zones of the removable overdenture at 'mastication': (**a**) – (**d**) – loading on the tooth No. 1–4, respectively (according to Figure 9, **a**).

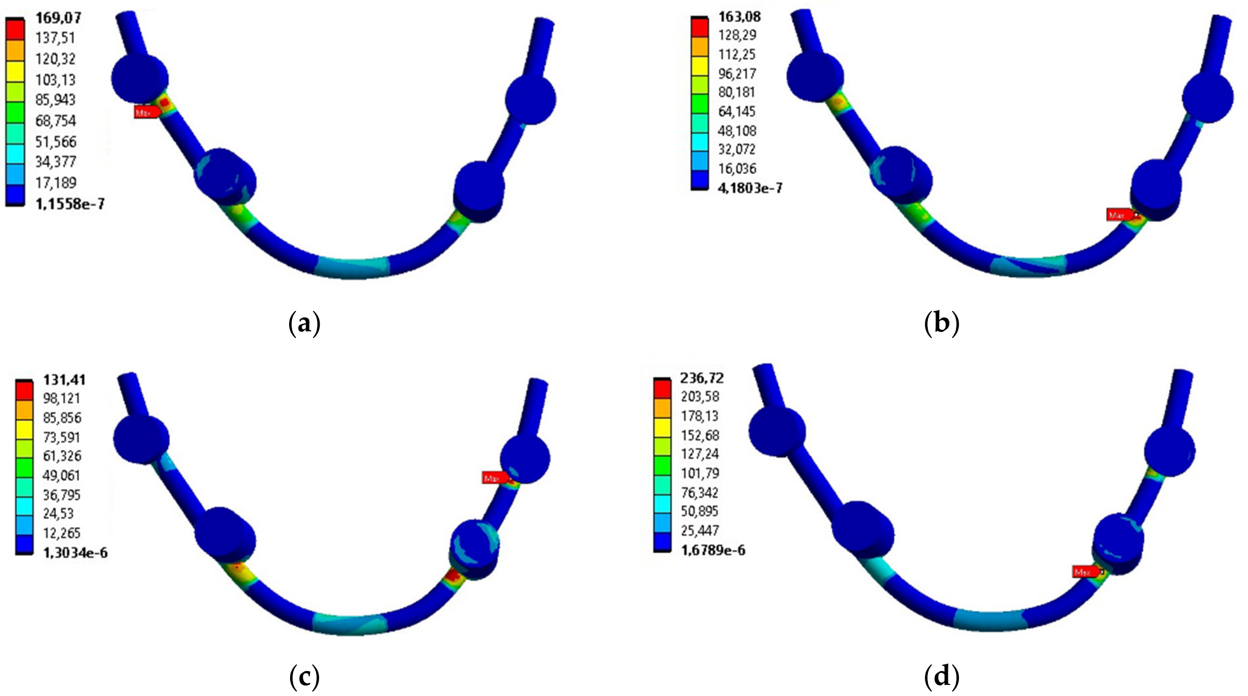

(**a**)–(**d**) – loading on the tooth No.1–4, respectively (according to Figure 9)

Figure S5. The equivalent stress distributions (MPa) in the splinting bar according to the results of computer simulation; the mastication loading.

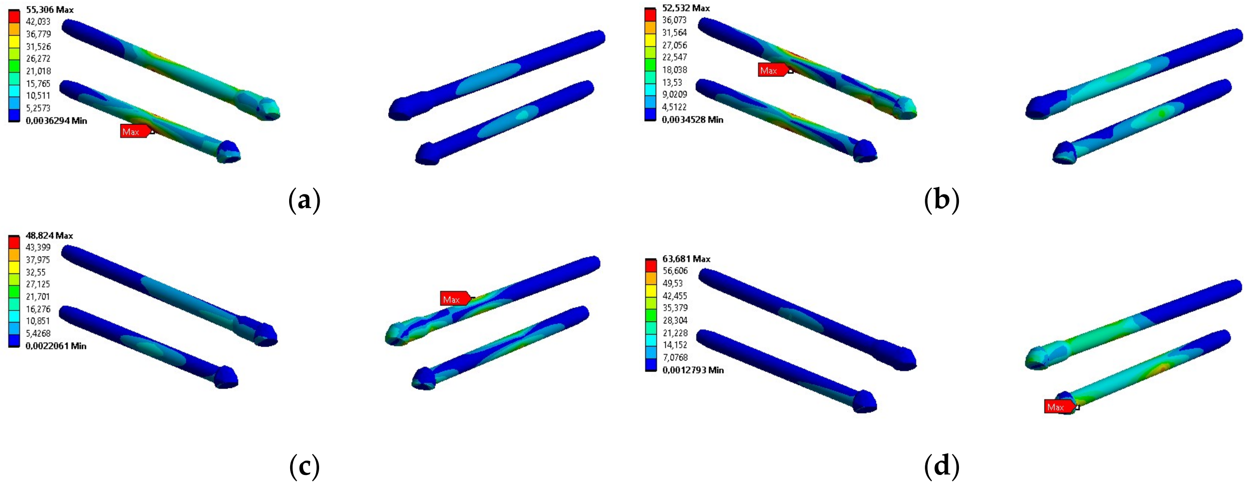

(**a**)–(**d**) – loading on the tooth No.1–4, respectively (according to Figure 9)

Figure S6. The equivalent stress distributions (MPa) in the implants according to the results of computer simulation; the mastication loading.

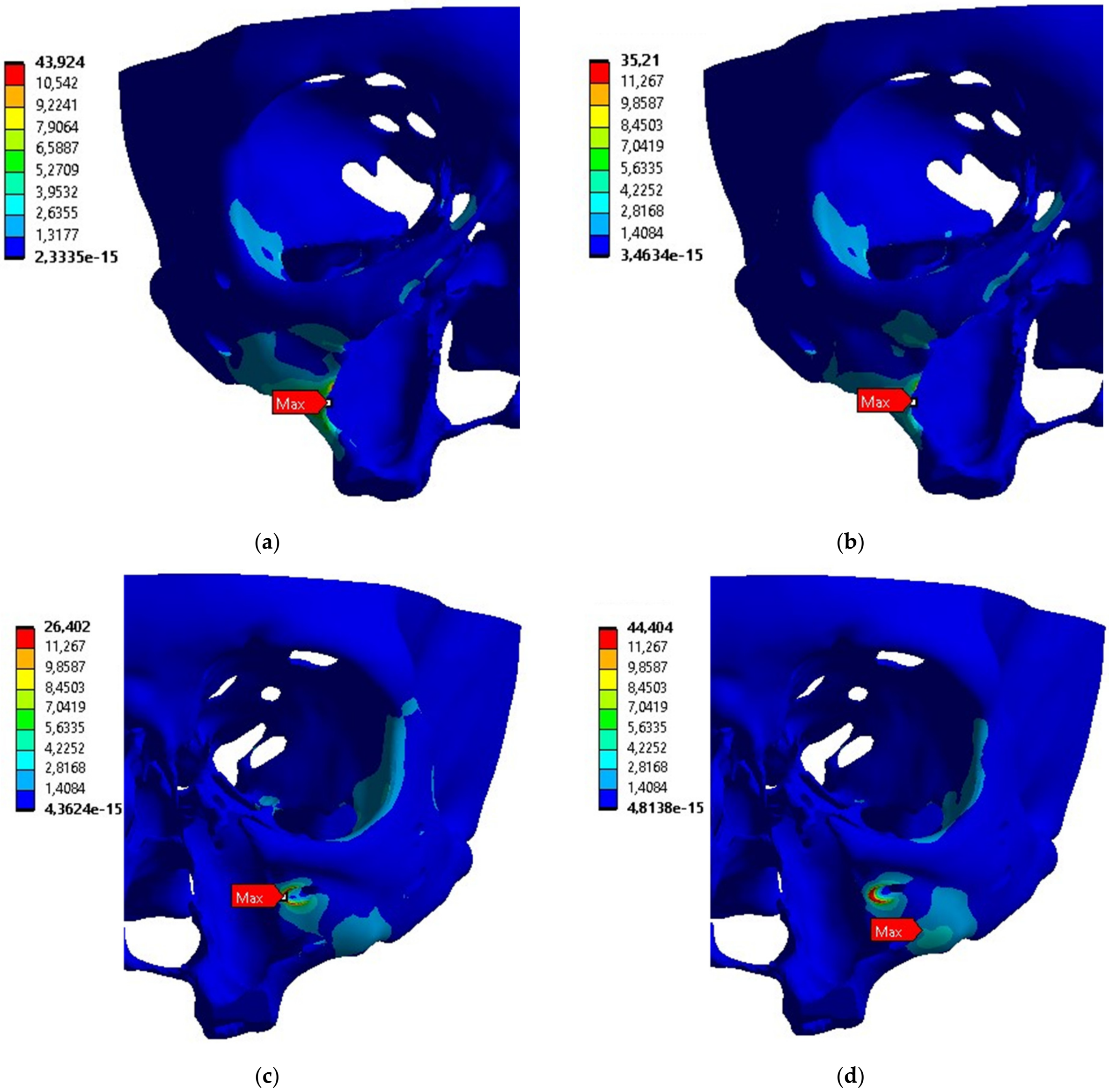

(**a**)–(**d**) – loading on the tooth No.1–4, respectively (according to Figure 9)

Figure S7. The equivalent stress distributions (MPa) in the zygomatic bones according to the results of computer simulation;f the mastication loading

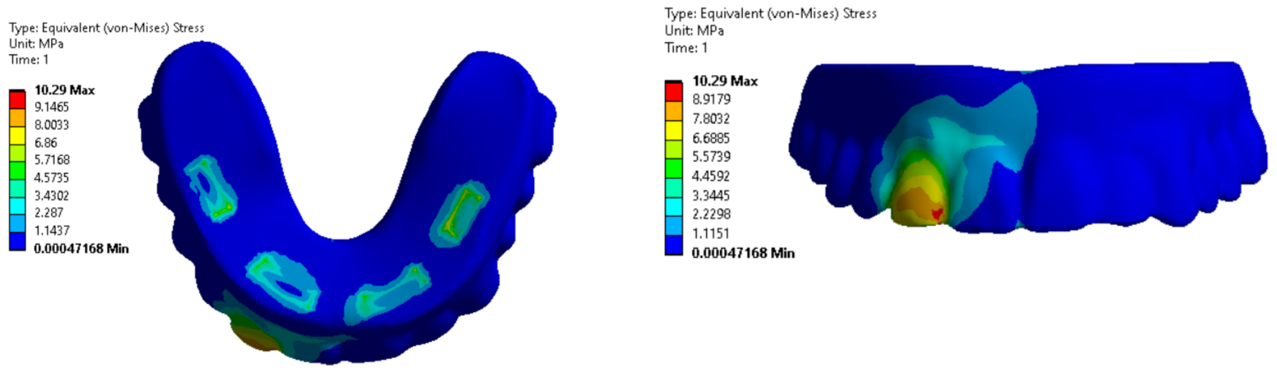

(**a**)

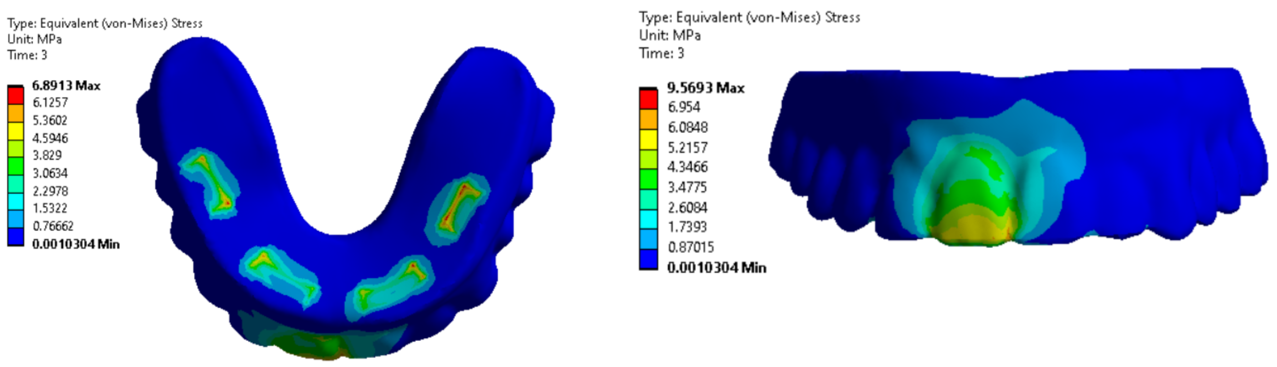

(**b**)

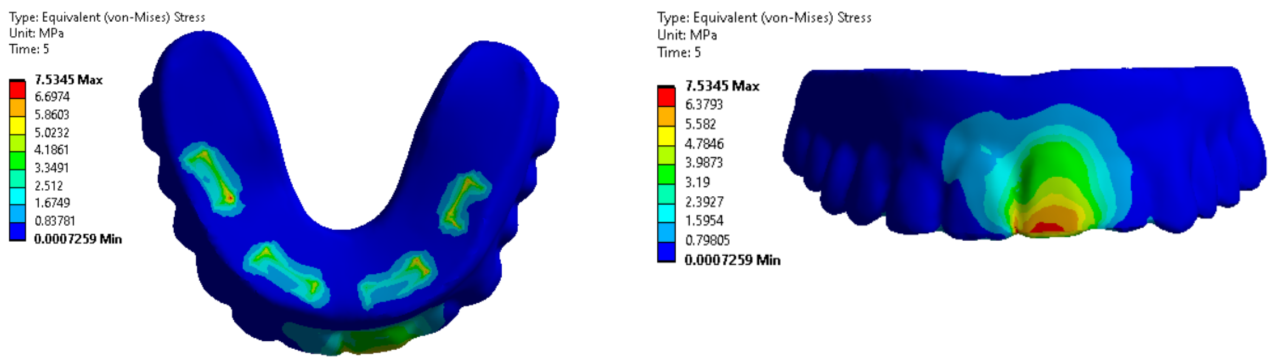

(**c**)

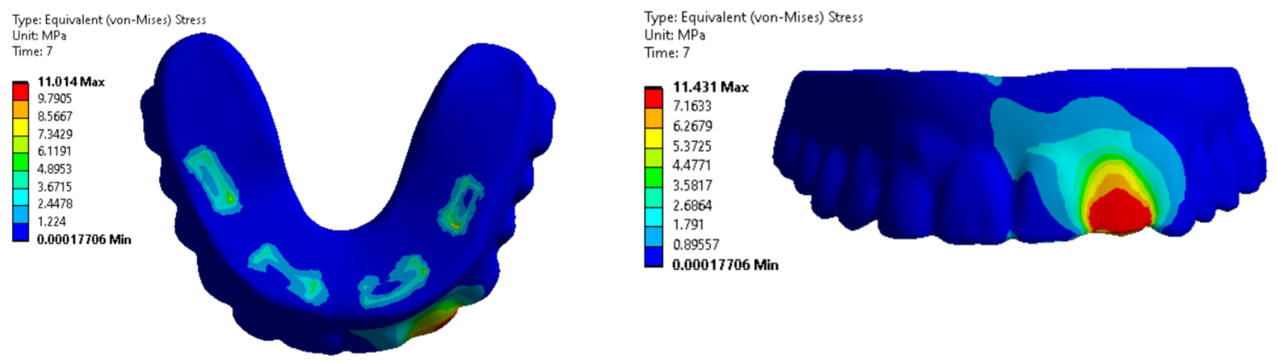

(**d**)

(**a**)–(**d**) – loading on the tooth No.1–4, respectively (according to Figure 12**, b**)

Figure S8. The equivalent stress distributions (MPa) in the removable overdenture according to the results of computer simulation; the of 90 ° biting loading

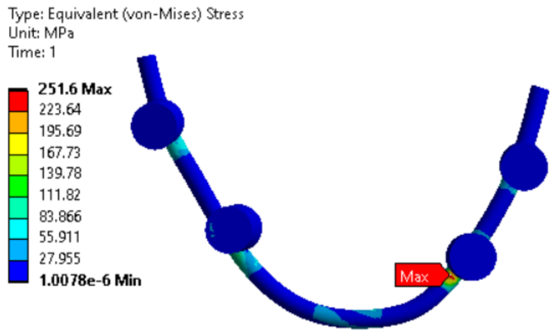

(**a**)

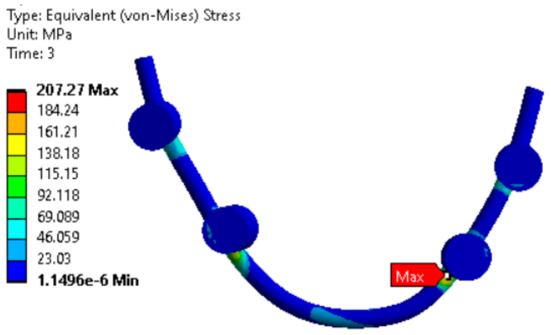

(**b**)

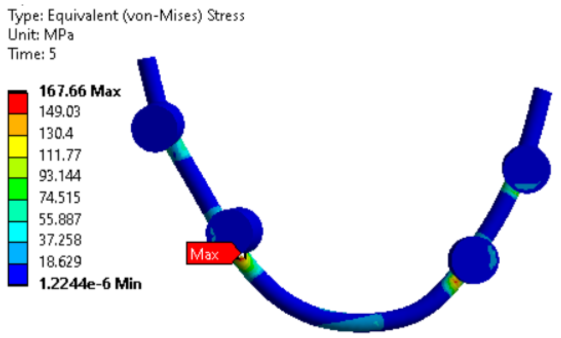

(**c**)

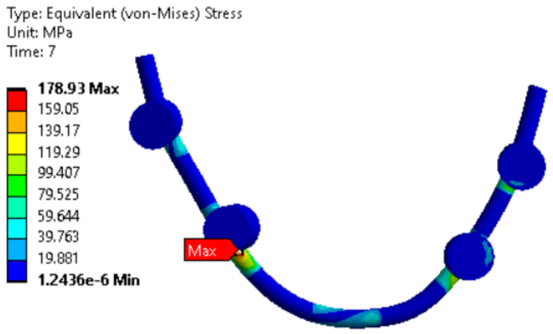

(**d**)

(**a**)–(**d**) loading on the tooth No.1–4, respectively (according to Figure 12**, b**)

Figure S9. The equivalent stress distributions (MPa) in the splinting bar according to the results of computer simulation; the 90 °biting loading

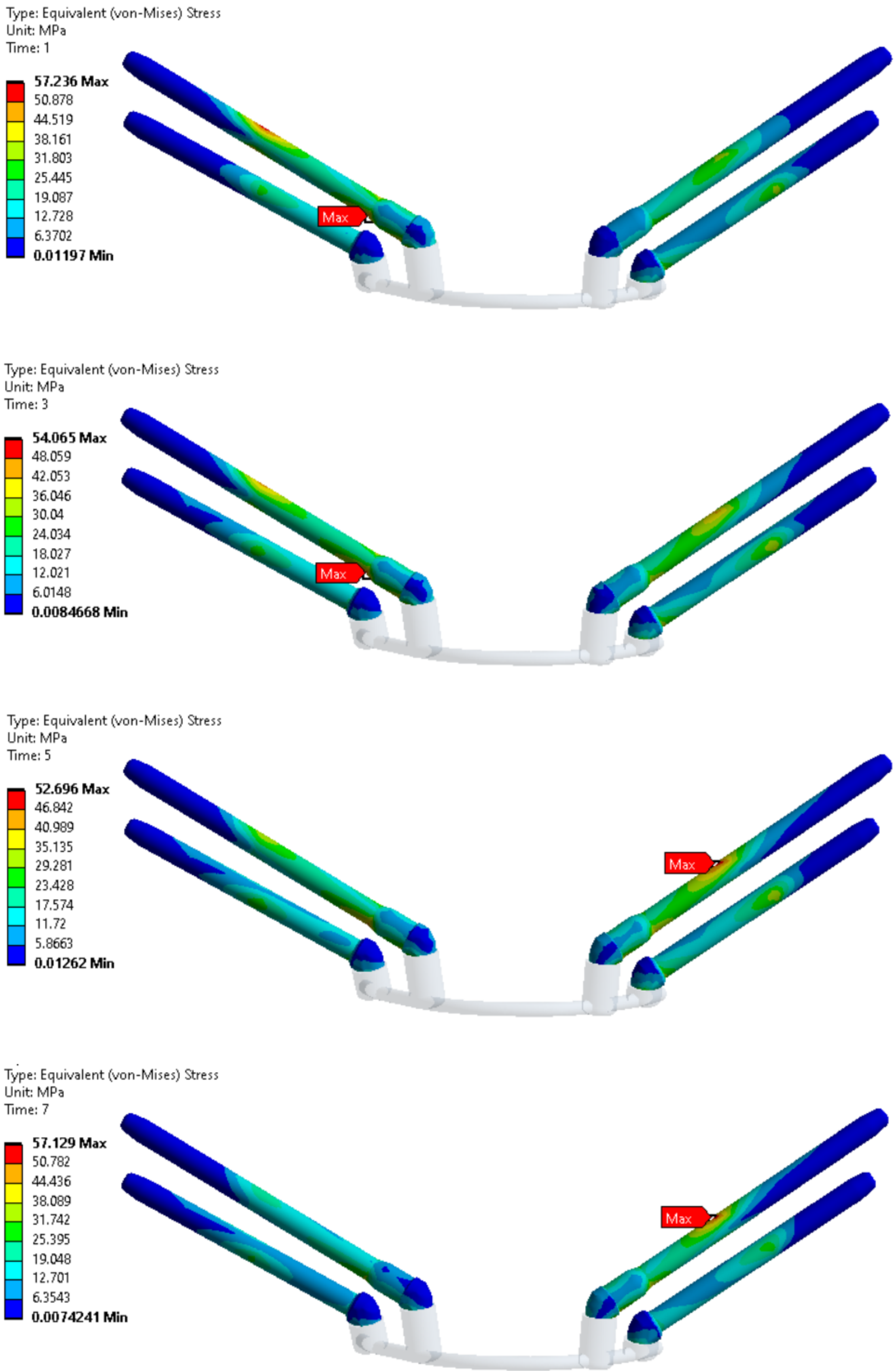

(**a**)

(**b**)

(**c**)

(**d**)

(**a**)–(**d**) loading on the tooth No.1–4, respectively (according to Figure 12**, b**)

Figure S10. The equivalent stress distributions (MPa) in the implants according to the results of computer simulation; the 90 °biting loading

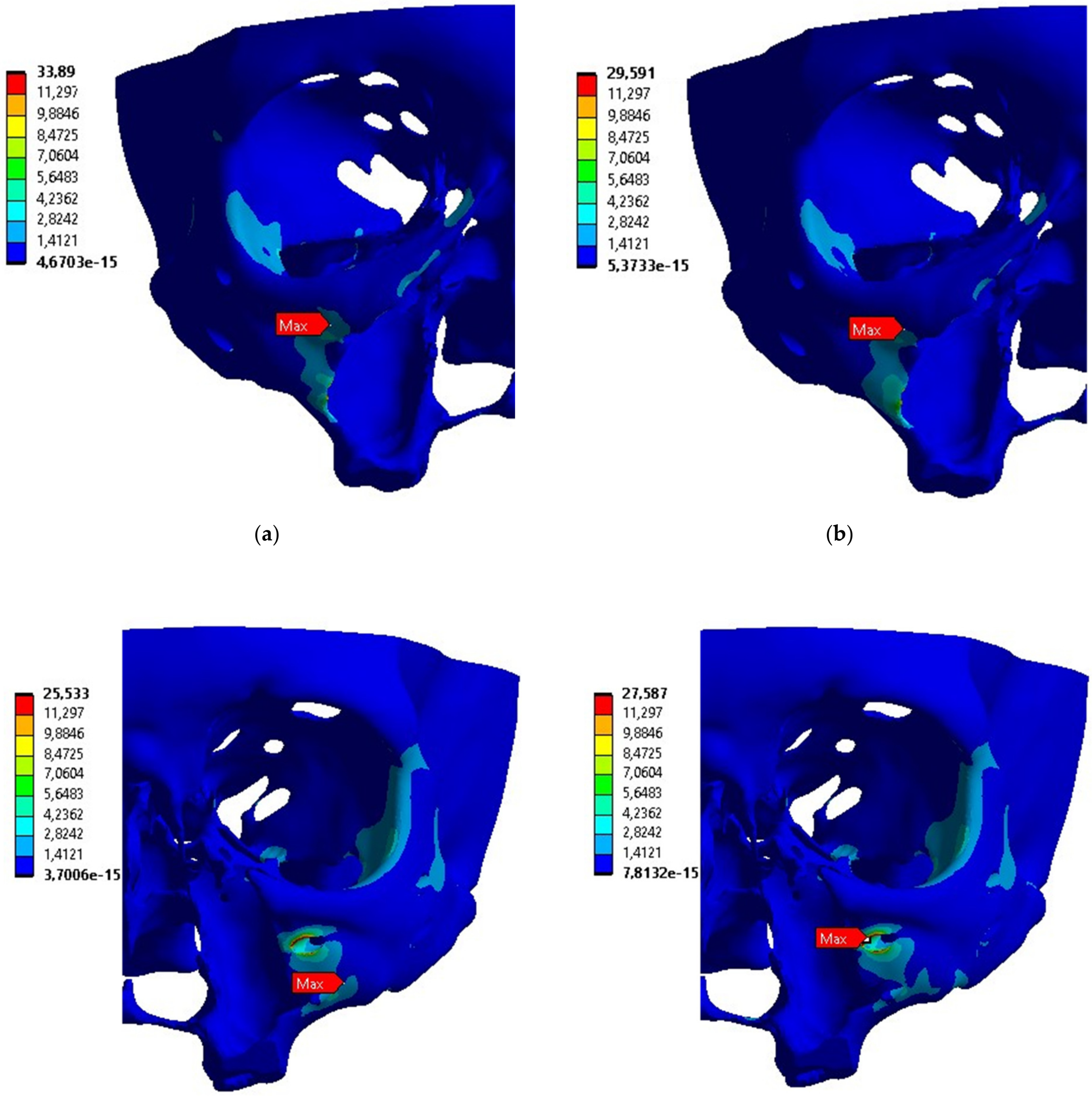

(**a**) (**b**)

(**c**) (**d**)

(**a**)–(**d**) loading on the tooth No.1–4, respectively (according to Figure 12**, b**)

Figure S11. The equivalent stress distributions (MPa) in the zygomatic bones according to the results of computer simulation; the 90 ° biting loading

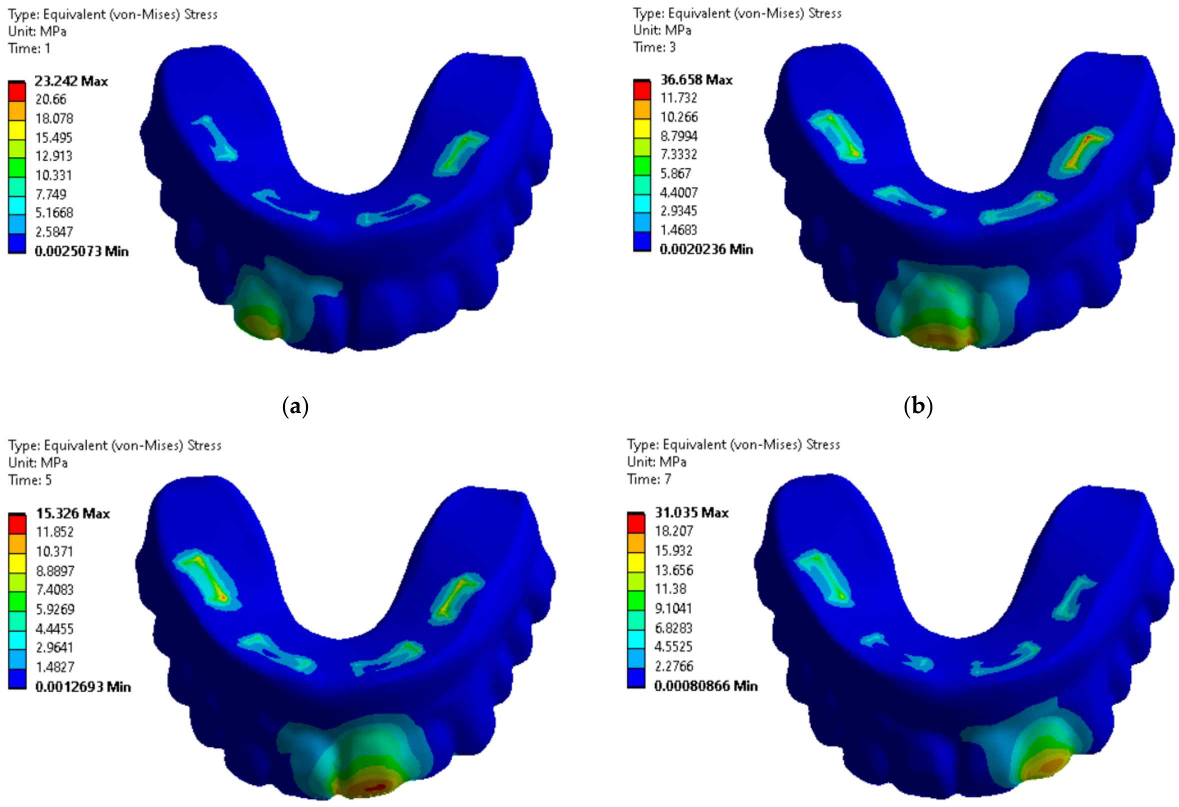

(**a**) (**b**)

(**c**) (**d**)

(**a**)–(**d**) loading on the tooth No.1–4, respectively (according to Figure 12, **c**)

Figure S12. The equivalent stress distributions (MPa) in the removable overdenture according to the results of computer simulation; the 45 ° biting loading

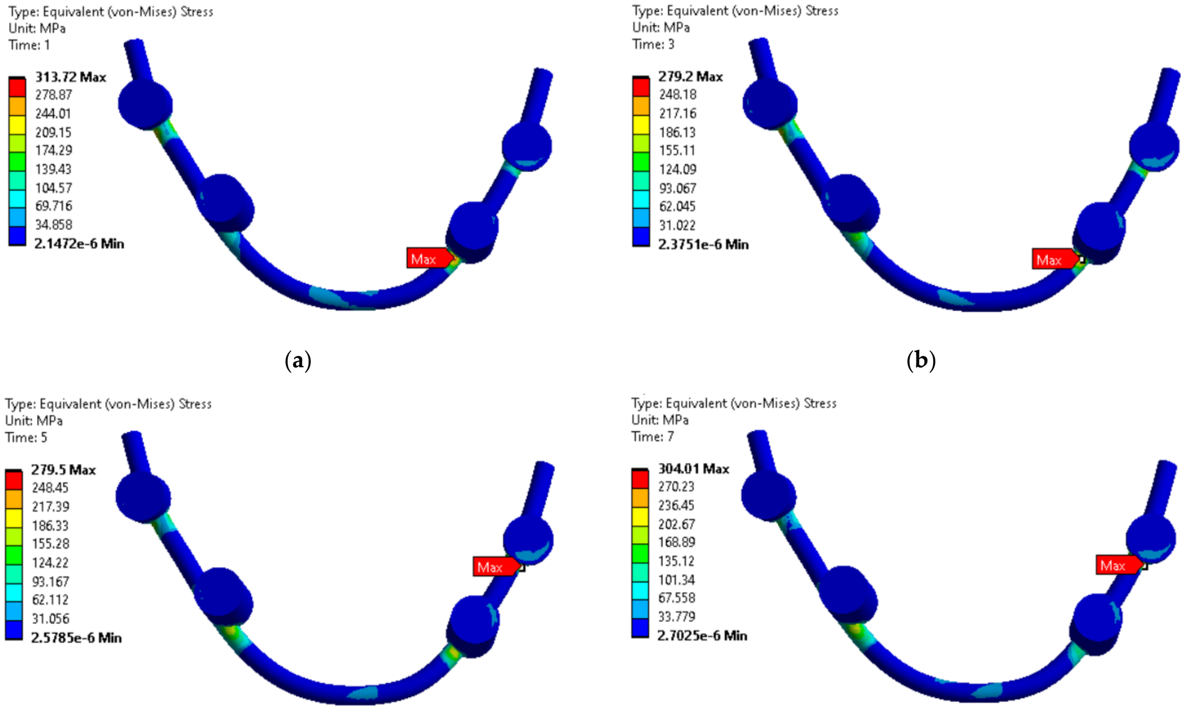

(**a**) (**b**)

(**c**) (**d**)

(**a**)–(**d**) – loading on the tooth No.1–4, respectively (according to Figure 12, **c**)

Figure S13. The equivalent stress distributions (MPa) in the splinting bar according to the results of computer simulation; the 45 ° biting loading

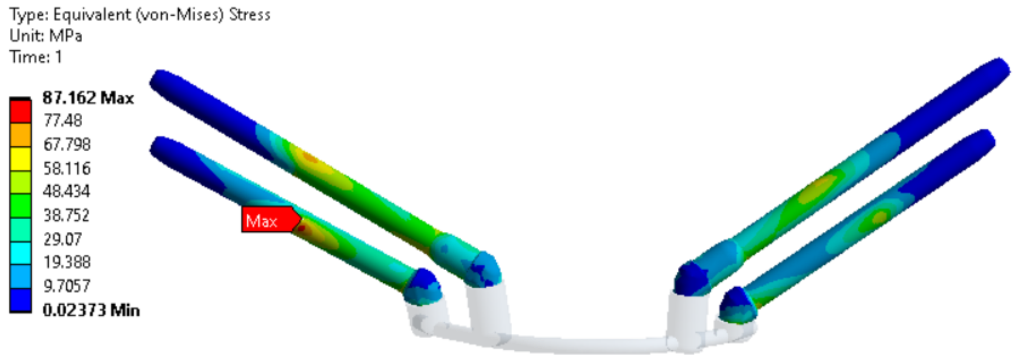

(**a**)

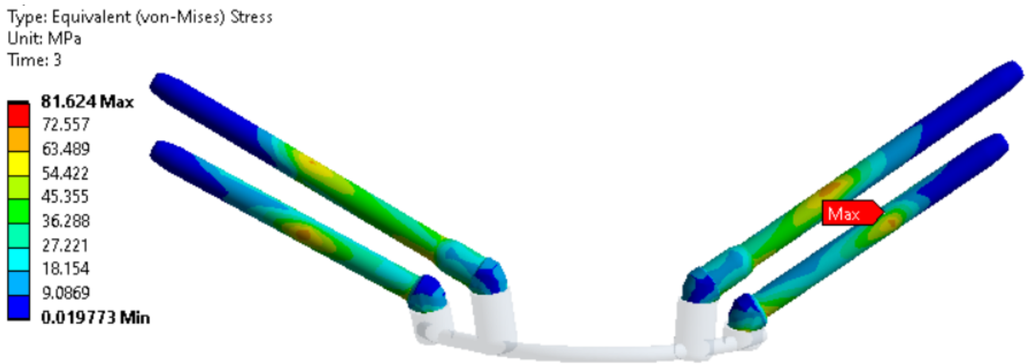

(**b**)

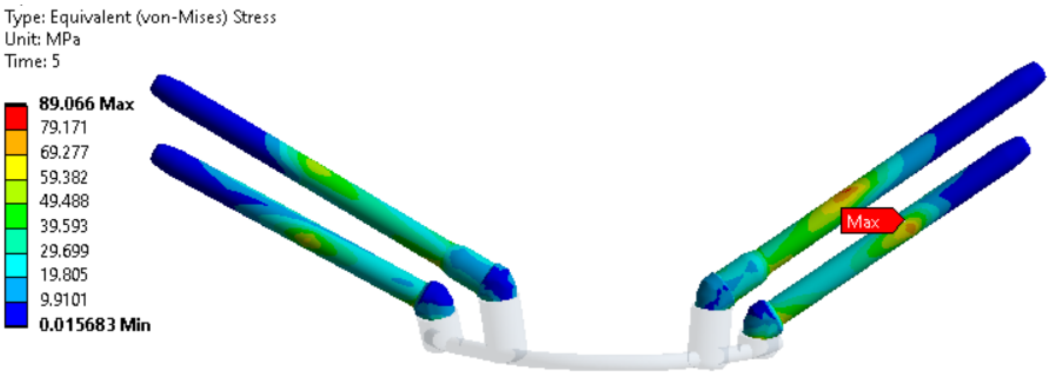

(**c**)

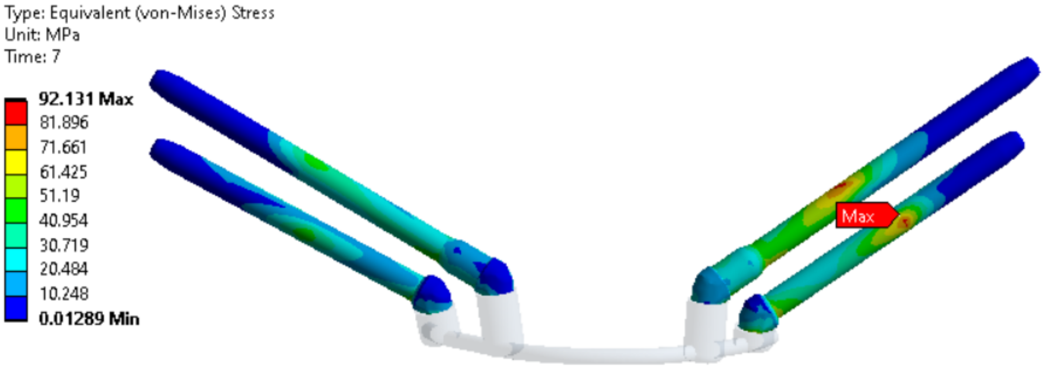

(**d**)

(**a**)–(**d**) loading on the tooth No.1–4, respectively (according to Figure 12**, c**)

Figure S14. The equivalent stress distributions (MPa) in the implants according to the results of computer simulation; the 45 ° biting loading

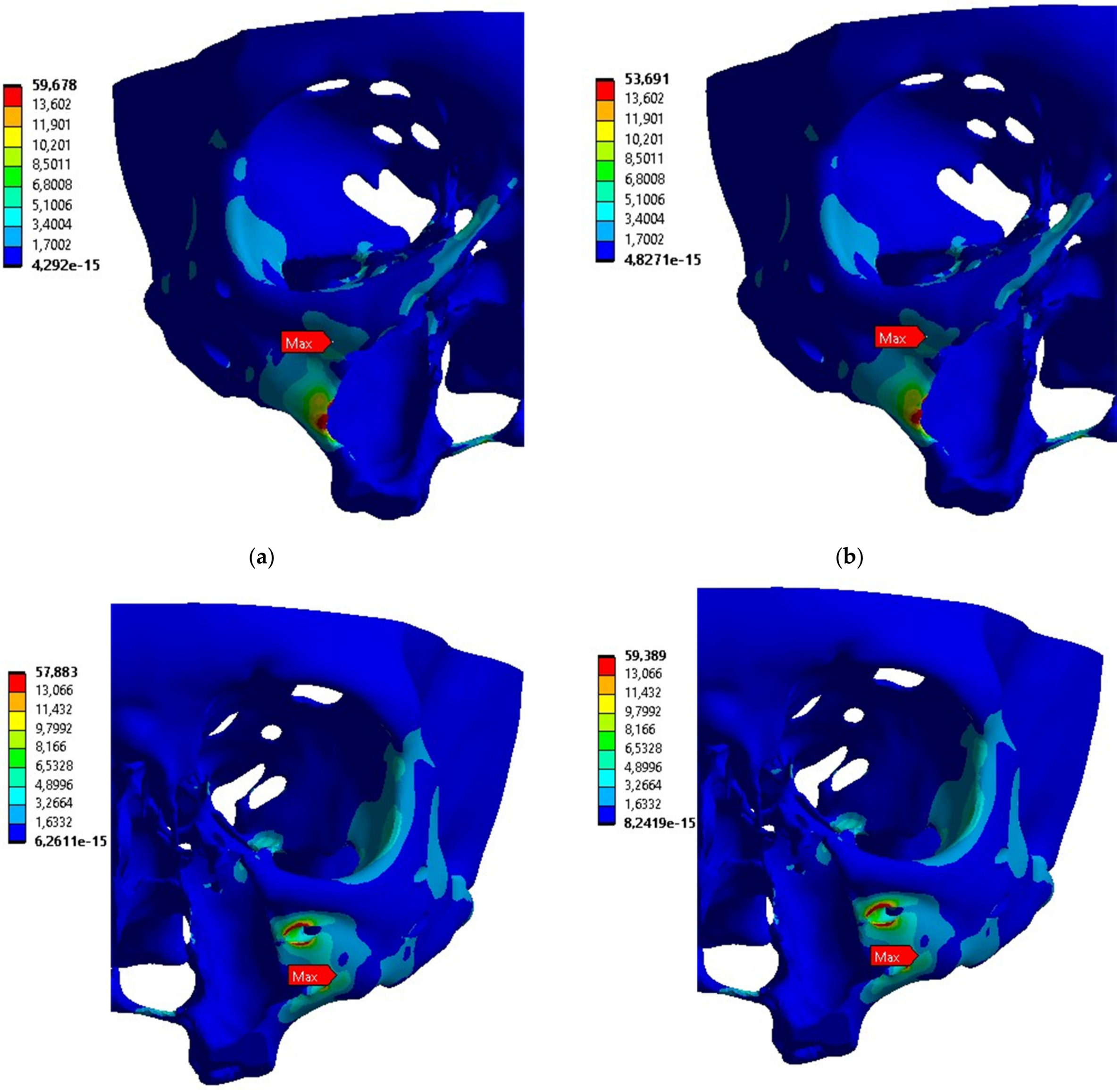

(**a**) (**b**)

(**c**) (**d**)

(**a**)–(**d**) loading on the tooth No.1–4, respectively (according to Figure 12**, c**)

Figure S15. The equivalent stress distributions (MPa) in the zygomatic bones according to the results of computer simulation; the 45 ° biting loading

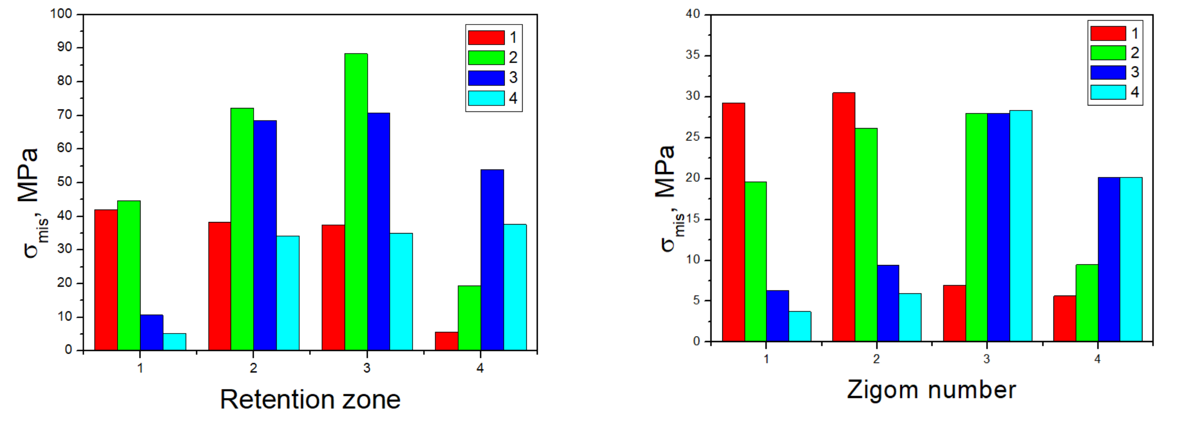

(**a**) (**b**)

Figure S16. The histograms of the distributions of the maximum equivalent stresses (MPa) in the elements of the dental structure during serial insertion/removal of the detachable overdenture; (**a**) splinting bar; (**b**) implant number.

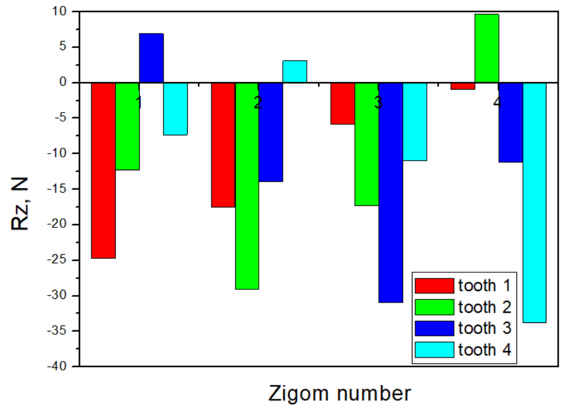

Figure S17. The histogram, plotted using the data presented in Table 2, characterizing the redistributions of reactive forces (N) in the bases [30] of the implants when applying the variable loads under mastication.

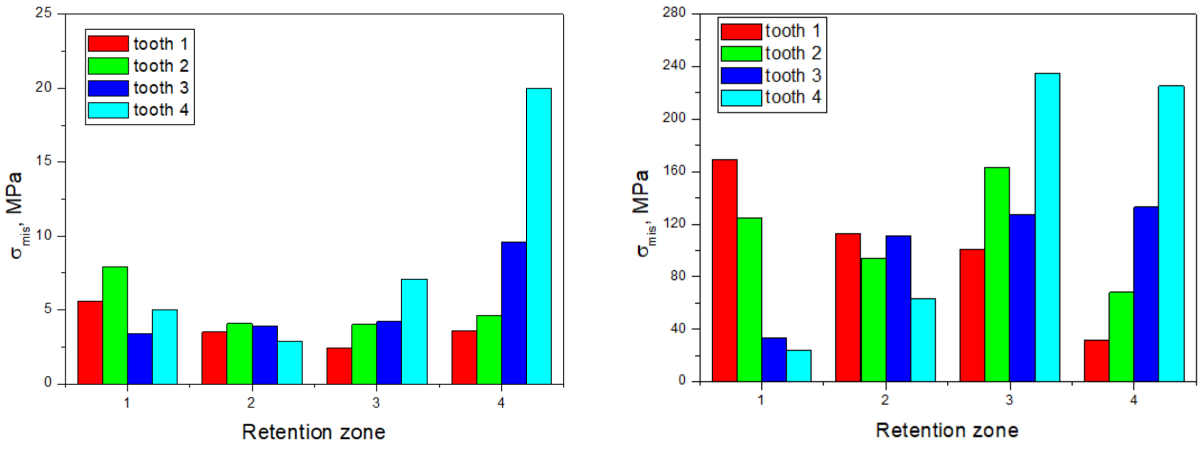

(**a**) (**b**)

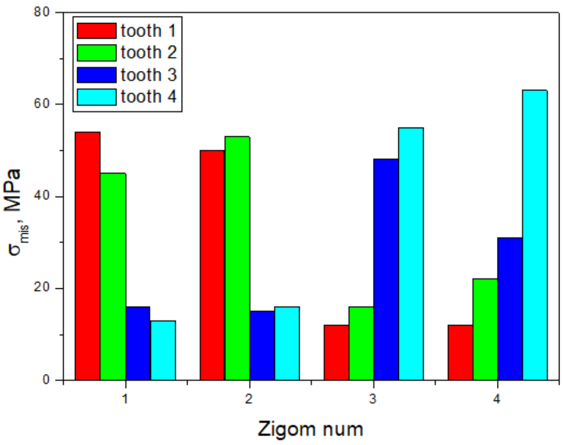

(**c**)

Figure S18. The histograms, plotted using the data presented in Table S2, reflecting the distributions of the maximum equivalent stresses in the components of the dental structure under mastication: (**a**) removable overdenture; (**b**) splinting bar; (**c**) implants.

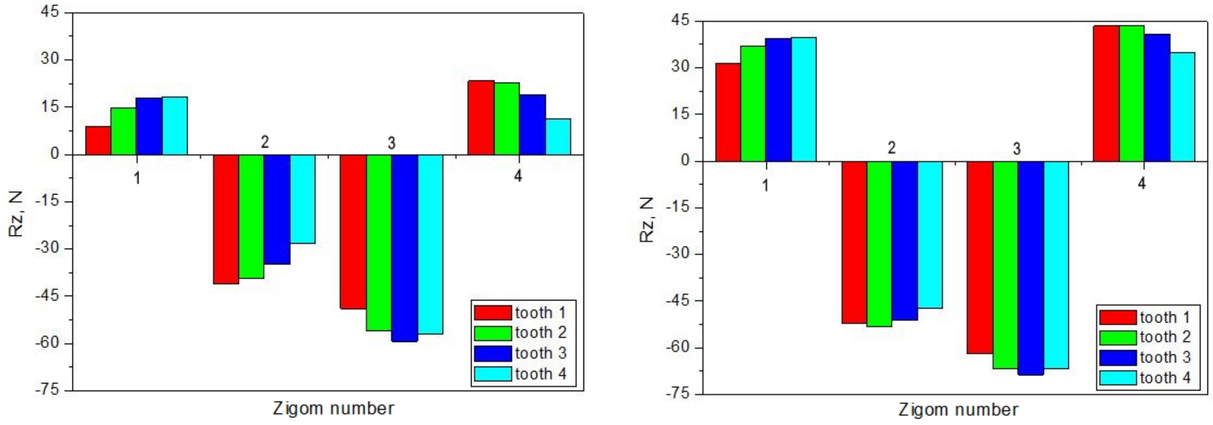

(**a**) (**b**)

Figure S19. The histograms of the redistributions of reactive forces (N) in the implants upon loading on the artificial incisors at the angles of 90 ° (**a**) and 45 ° (**b**).

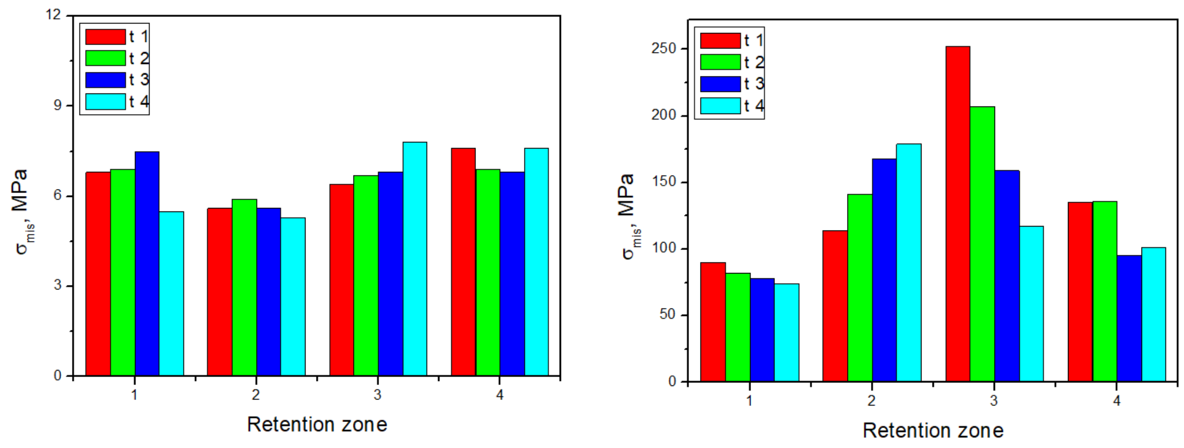

(**a**) (**b**)

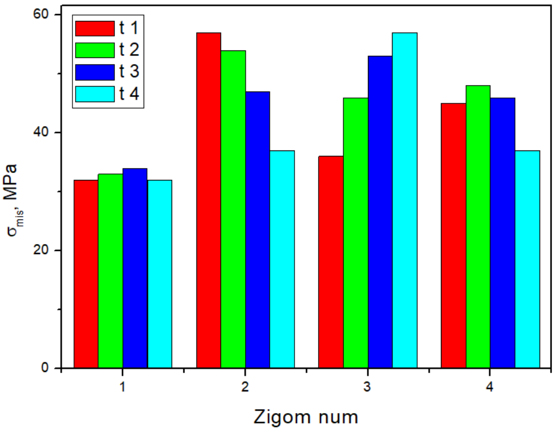

(**c**)

Figure S20. The histograms plotted using the data presented in Table S3, reflecting the distributions of the maximum equivalent stresses in the components of the dental structure at 90 ° anterior biting: (**a**) removable denture; (**b**) splinting bar; (**c**) implants.

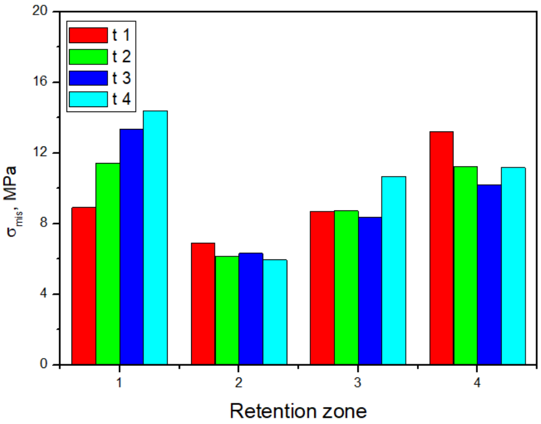

(**a**)

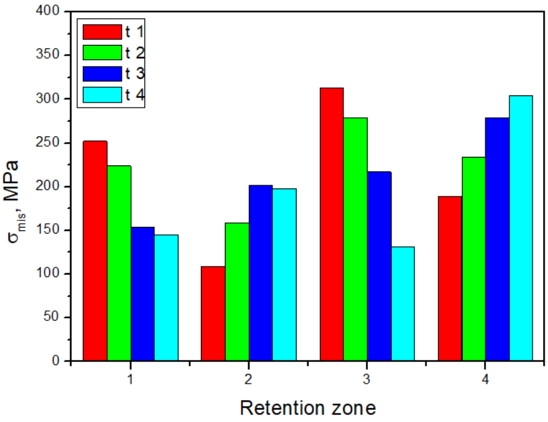

(**b**)

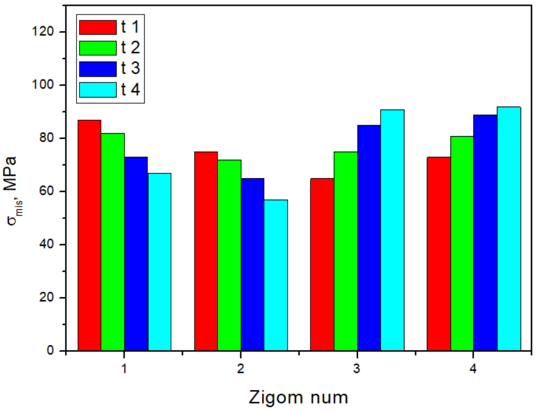

(**c**)

Figure S21. The histograms plotted using the data presented in Table S4, reflecting the distributions of the maximum equivalent stresses in the components of the dental structure at 45 ° anterior biting: (**a**) removable overdenture; (**b**) splinting bar; (**c**) implants.
